# The SAGA histone acetyltransferase module targets SMC5/6 to specific genes

**DOI:** 10.1186/s13072-023-00480-z

**Published:** 2023-02-16

**Authors:** L. Mahrik, B. Stefanovie, A. Maresova, J. Princova, P. Kolesar, E. Lelkes, C. Faux, D. Helmlinger, M. Prevorovsky, J. J. Palecek

**Affiliations:** 1 National Centre for Biomolecular Research, Faculty of Science, Masaryk University, Kotlarska 2, 61137 Brno, Czech Republic; 2 Mendel Centre for Plant Genomics and Proteomics, Central European Institute of Technology, Masaryk University, Kamenice 5, 62500 Brno, Czech Republic; 3 Department of Cell Biology, Faculty of Science, Charles University, Vinicna 7, 12800 Prague, Czech Republic; 4 Centre de Recherche en Biologie Cellulaire de Montpellier, University of Montpellier, CNRS, 1919 Route de Mende, 34293 Montpellier Cedex 05, France; 5 National Centre for Biomolecular Research, Faculty of Science, Masaryk University, Kamenice 5, 62500 Brno, Czech Republic

**Keywords:** Genetic and protein–protein interactions, SMC5/6 complex targeting, Nse3 KITE, SAGA histone acetyltransferase module, Gcn5, Ada2, Histone H3K9ac acetylation, Chromatin accessibility, DNA repair, rDNA, Gene regions

## Abstract

**Background:**

Structural Maintenance of Chromosomes (SMC) complexes are molecular machines driving chromatin organization at higher levels. In eukaryotes, three SMC complexes (cohesin, condensin and SMC5/6) play key roles in cohesion, condensation, replication, transcription and DNA repair. Their physical binding to DNA requires accessible chromatin.

**Results:**

We performed a genetic screen in fission yeast to identify novel factors required for SMC5/6 binding to DNA. We identified 79 genes of which histone acetyltransferases (HATs) were the most represented. Genetic and phenotypic analyses suggested a particularly strong functional relationship between the SMC5/6 and SAGA complexes. Furthermore, several SMC5/6 subunits physically interacted with SAGA HAT module components Gcn5 and Ada2. As Gcn5-dependent acetylation facilitates the accessibility of chromatin to DNA-repair proteins, we first analysed the formation of DNA-damage-induced SMC5/6 foci in the Δ*gcn5* mutant. The SMC5/6 foci formed normally in Δ*gcn5,* suggesting SAGA-independent SMC5/6 localization to DNA-damaged sites. Next, we used Nse4-FLAG chromatin-immunoprecipitation (ChIP-seq) analysis in unchallenged cells to assess SMC5/6 distribution. A significant portion of SMC5/6 accumulated within gene regions in wild-type cells, which was reduced in Δ*gcn5* and Δ*ada2* mutants. The drop in SMC5/6 levels was also observed in *gcn5*-E191Q acetyltransferase-dead mutant.

**Conclusion:**

Our data show genetic and physical interactions between SMC5/6 and SAGA complexes. The ChIP-seq analysis suggests that SAGA HAT module targets SMC5/6 to specific gene regions and facilitates their accessibility for SMC5/6 loading.

**Supplementary Information:**

The online version contains supplementary material available at 10.1186/s13072-023-00480-z.

## Background

Chromatin is composed of DNA and protein complexes structured at multiple levels to ensure its spatial and functional organization [1]. Histone proteins pack DNA into nucleosomes and their arrays at the basic level. At the higher levels, structural maintenance of chromosomes (SMC) complexes (cohesin, condensin and SMC5/6) assist in the formation of high-order structures like topologically associated domains or condensed mitotic chromosomes [2]. Chromatin compaction affects DNA accessibility at each level. Histone chaperones, modifiers, and remodelers can loosen, move or remodel nucleosomes to modulate essential processes like transcription or DNA repair [3, 4]. For example, the histone-modifying SAGA complex acetylates H3 histones at promoter regions, contributing to chromatin opening and facilitating the assembly of transcription initiation complexes onto core promoters and the recruitment of factors that directly interact with DNA [5].

The SMC complexes play roles in all key chromatin processes, including cohesion, condensation, replication, transcription and DNA repair. Their cores comprise the long-armed Smc, kleisin and kleisin-associated (KITE or HAWK) subunits [6, 7]. Uniquely, SMC5/6 complexes contain the highly conserved Nse1 [8] and Nse2 ubiquitin- and SUMO-ligases, respectively [9, 10]. The Smc subunits are primarily built of head ATPase domains, long anti-parallel coiled-coil arms and hinges [11–13]. Two Smc molecules form stable dimers via their hinge domains, and without ATP, their arms align into rod-like structures [14, 15]. The binding of ATP molecules to the ATPase head domains promotes the formation of large annular structures [16, 17]. The ATP binding−hydrolysis cycle drives ring-to-rod dynamic changes and promotes DNA translocation or loop extrusion [18–21].

SMC complexes were believed to interact only topologically with chromatin fibres via their large ring-shaped structures, which can embrace and traverse large chromatin complexes, including nucleosomes [13]. However, growing evidence suggests their requirement for open chromatin and direct physical binding to DNA [22–24]. Moreover, Piazza et al. [22] described the preferential binding of condensin’s kleisin-associated HAWK subunits to free DNA over nucleosomal DNA. Recent cryoEM analyses showed the formation of K-compartments within all SMC complexes, consisting of ATP-bound Smc heads, kleisin and kleisin-associated subunits, which can accommodate only free DNA [17, 25–27]. In line with these findings, the SAGA complex assists in loading condensin at open chromatin regions of highly transcribed genes in fission yeast [24]. Similarly, the RSC chromatin remodelling complex recruits the Scc2-Scc4 factor to nucleosome-free regions, assisting in cohesin loading at these sites [28–30].

Recently, it was shown that the SMC5/6 K-compartment, composed of Smc5-Smc6 heads, Nse4 kleisin and Nse1−Nse3 kleisin-associated KITE subunits, binds free DNA [17, 22]. In our previous study, we characterized the binding of the C-terminal winged-helix (WHB) domain of Nse3 to DNA and described the essential role of Nse3-DNA interaction for SMC5/6 loading or accumulation [22]. Here, we performed a genetic screen with a *nse3-R254E* fission yeast mutant that exhibits reduced DNA-binding affinity to identify new factors required for its viability. We found strong genetic interactions with the SAGA and NuA4 histone acetyltransferase (HAT) complexes. Using chromatin-immunoprecipitation (ChIP-seq) analysis, we observed a significant portion of SMC5/6 accumulated within gene regions, which was reduced in SAGA HAT deletion (Δ*ada2* and Δ*gcn5*) mutants. The magnitude of the decrease in SMC5/6 occupancy correlated with the SAGA-modified H3K9ac levels around the transcription start sites. The SMC5/6 reduced levels were also observed in *gcn5*-*E191Q* acetyltransferase-dead mutant, suggesting that the SAGA HAT module may target SMC5/6 to gene regions and facilitate the accessibility of chromatin for SMC5/6 loading.

## Results

### Genetic screen with a DNA-binding defective allele of the SMC5/6 complex

To identify factors that facilitate the loading or accumulation of the SMC5/6 complex on chromatin, we performed a genetic search for genes affecting the survival of cells with compromised DNA-binding ability of the SMC5/6 complex [22]. First, we created a query fission yeast strain with the DNA-binding defective *nse3-R254E* mutation in the PEM2 background (Additional file 1: Fig. S1A; [31]) and crossed it against the whole gene deletion yeast collection from BIONEER [32]. Using yeast colony size phenotypic readout [33], we identified 79 deletion strains that exhibited a negative genetic interaction with *nse3-R254E* (Additional file 1: Table S1).

**Fig. 1:**
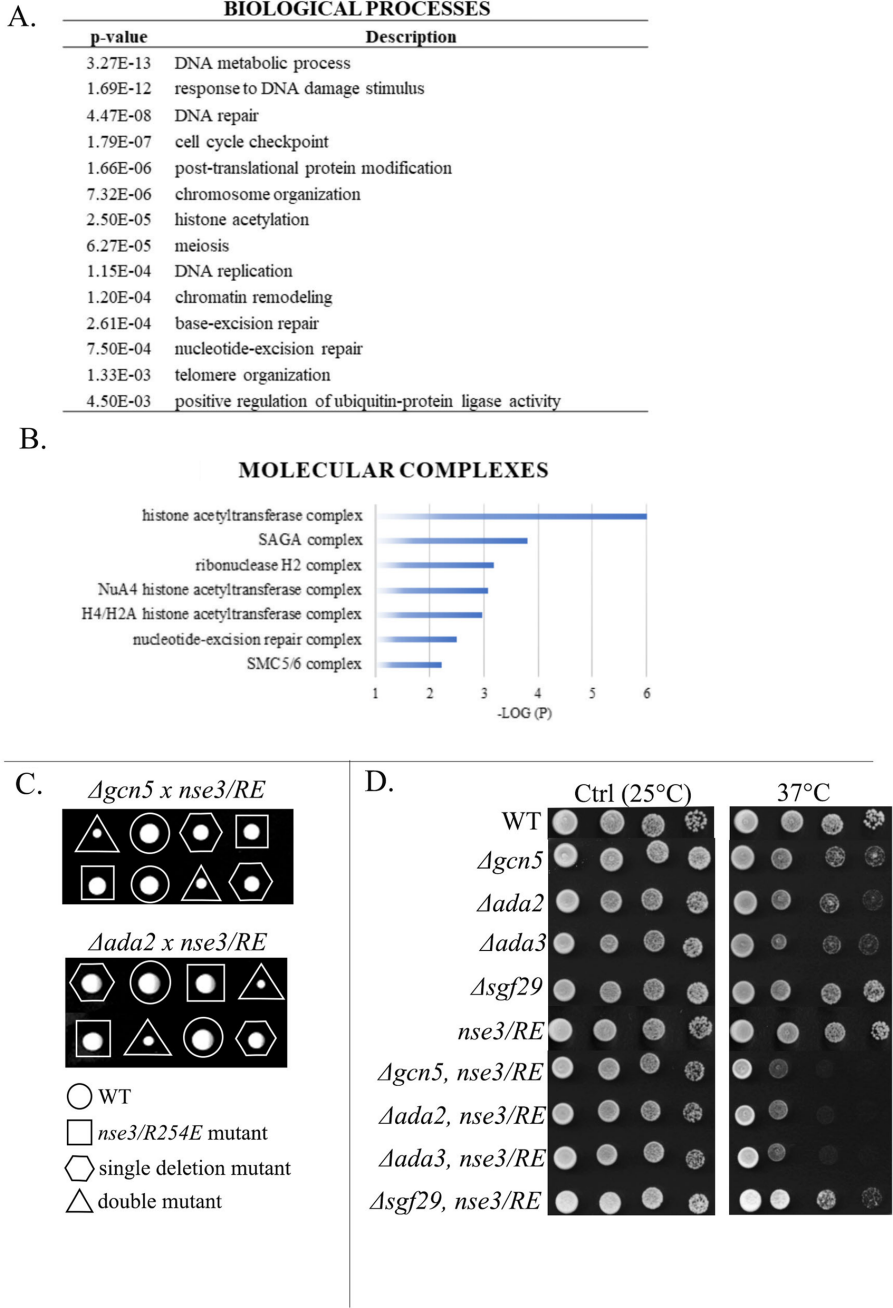
Strong genetic relation between the SMC5/6 and histone acetyltransferases. **A** Summary of significantly enriched GO categories for Biological processes of genes with genetic interactions with *nse3*-*R254E* (*p* < 0.005). **B** Negative log10 (*p*-values) evaluating the significance of the main GO Cellular component terms identified in the set of the 79 genes. Only the molecular complexes are shown. **C** Tetrad analysis of the heterozygous diploid fission yeast strains. The colony size of the *nse3-R254E*, Δ*gcn5* and *nse3-R254E*, Δ*ada2* double mutant is significantly reduced (triangle). Single and double mutant alleles are indicated. **D** Ten-fold serial dilutions of the indicated strains were plated onto YES media and grown at 25 °C (control) or 37 °C. At least three independent drop tests have been carried out, and one of them is displayed as representative. The *nse3-R254E* (*nse3/RE*) mutation enhanced the sensitivity of the Δ*ada2*, Δ*ada3* and Δ*gcn5* mutants to the higher temperature

To validate our results, we randomly selected 19 of the 79 strains and crossed them with the original *nse3-R254E* strain [22]. Tetrad analysis confirmed that these mutations are synthetically sick or lethal with the *nse3-R254E* mutation (Additional file 1: Fig. S1B). Analysis of all 79 interacting genes for the Biological process category using Gene ontologies (GO; [34]) showed the highest scores for DNA repair, chromatin organization, meiosis and replication processes (Fig. 1A and Additional file 1: Table S2), in line with previous studies (reviewed in [35, 36]). Reassuringly, several *nse3-R254E* genetic interactions overlapped with the genetic interactions of other *smc5/6* mutants [22, 37–42], supporting the validity of our screen results.

### Genetic interactions between SMC5/6 and histone acetyltransferase complexes

The analysis of the Cellular components GO of the 79 interacting genes showed the highest scores for histone acetyltransferase (HAT) complexes (SAGA and NuA4; Fig. 1B and Additional file 1: Table S3). Three (Ada2, Ada3/Ngg1, Gcn5) out of four HAT module subunits and one (Ubp8) out of four DUB module subunits of the SAGA complex were identified as hits in our screen [5]. We verified their genetic interactions using tetrad analysis (Figs. 1C and S1B) and also tested the other non-essential SAGA subunits not detected in our screen. The mating defects of the Δ*ada1,* Δ*spt7,* Δ*spt8* and Δ*spt20* deletion mutants hampered the double mutant preparation [43]. However, the tetrad analysis of the other non-essential SAGA subunits (Additional file 1: Fig. S1C) showed negative genetic interactions with the *nse3-R254E* mutation (Additional file 1: Fig. S1D), suggesting a strong functional relationship between the SMC5/6 and SAGA complexes.

Interestingly, the temperature-sensitive (*ts*) phenotypes of the SAGA HAT module Δ*ada2*, Δ*ada3* and Δ*gcn5* mutants were enhanced by *nse3-R254E* (Fig. 1D; [43]). The *ts* phenotypes were also enhanced by the *smc6-74* and *smc6-X* hypomorphic mutations (Additional file 1: Fig. S2A; [38, 44]). We also used *nse1-R188E* and *nse2-SA* mutations, which specifically abrogate DNA-repair function (ubiquitin- and SUMO- ligase activity, respectively), but not the SMC5/6 essential function [8, 9]. However, these mutations did not affect the *ts* phenotype of Δ*gcn5* (Additional file 1: Fig. S2A; not shown). These data suggest that the SMC5/6 essential function supports cell survival at higher temperatures in the absence of the HAT module and, conversely, that the viability of the *nse3-R254E* mutant is compromised in the absence of a functional SAGA HAT module.

### SMC5/6 and SAGA physically interact

**Fig. 2:**
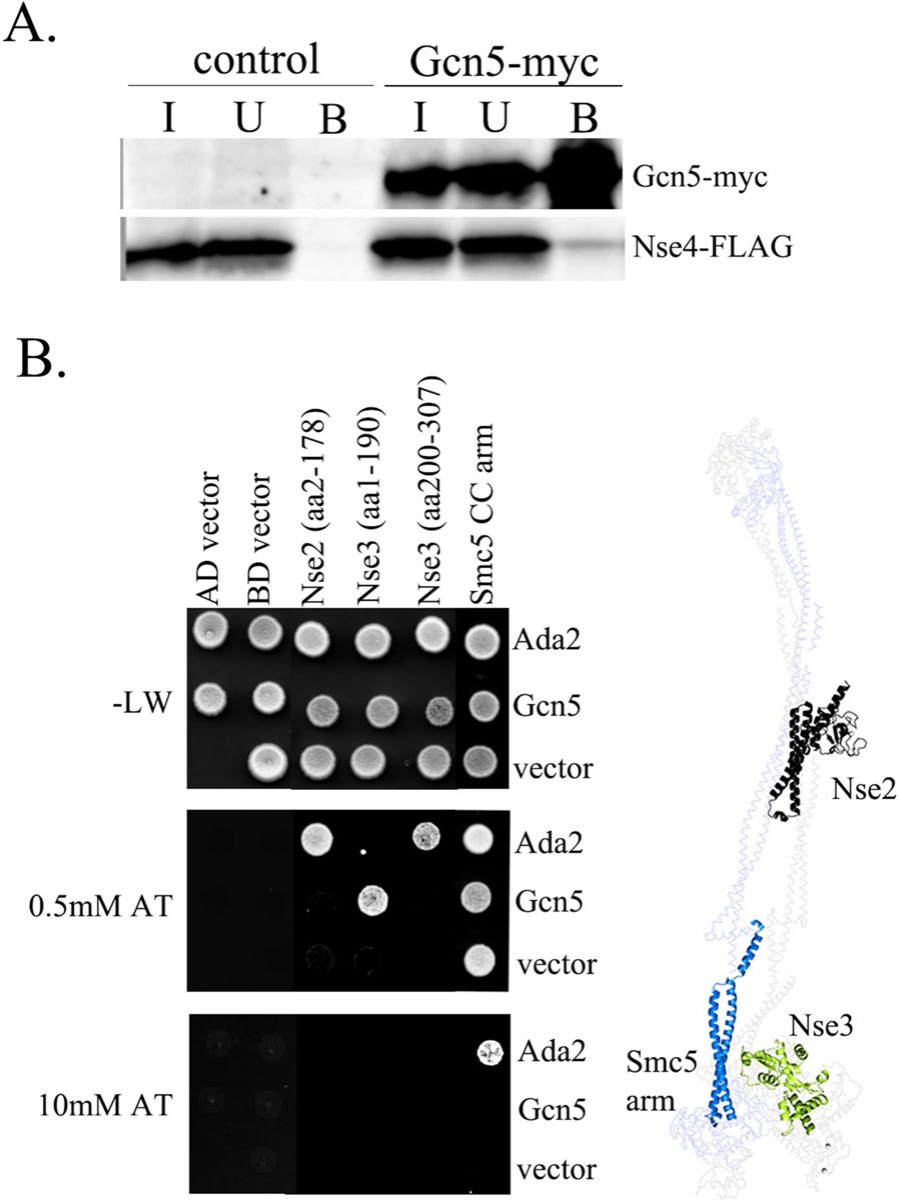
Interactions between the SMC5/6 and SAGA complexes. **A** Extracts from fission yeast strains MMP21 (Nse4-FLAG) and YLJ507 (Nse4-FLAG and Gcn5-myc) were immunoprecipitated using the anti-myc antibody. The input (I), unbound (U) and bound (B) fractions were separated by 12% SDS-PAGE. The Nse4-FLAG and Gcn5-myc proteins were analysed on a western blot using anti-FLAG-HRP and anti-myc-HRP, respectively. **B** The yeast two-hybrid (Y2H) system was used to determine individual protein–protein interactions between SMC5/6 and SAGA HAT module subunits. The Gal4AD or Gal4BD domains fused to the full-length Ada2 or Gcn5 subunits were co-transformed together with the fragments of SMC5/6 subunits into the PJ69 cells and grown on the plates without Leu, Trp (-L, W; control plates). The protein–protein interactions between SMC5/6 and SAGA were scored by the growth of the yeast PJ69 transformants on the plates without Leu, Trp and His, containing 3-Amino-1,2,4-triazole (0.5 mM AT or 10 mM AT plates). The fragments were as follows: Gal4BD-Nse2 (aa2-178), Gal4AD-Nse3 (aa1-190), Gal4AD-Nse3 (aa200-307) and Gal4BD-Smc5 CC arm (aa170-225 + 837-910). In control experiments, respective empty pGADT7 (AD) or pGBKT7 (BD) vector was co-transformed with either SAGA or SMC5/6 construct. Note that the Gal4BD-Smc5 CC arm construct self-activated (Smc5-vector combination) and was therefore grown on 10 mM AT plates to assess its binding to Ada2 (Smc5-Ada2 combination). The Nse2 (black), Nse3 (green) and Smc5 (blue) subunits binding either Ada2 or Gcn5 are highlighted within the SMC5/6 rod-shaped structural model (shaded; based on the 7QCD structure from [14]) next to the Y2H results

The strong genetic relationship between the SAGA HAT module and the SMC5/6 complex prompted us to test their mutual physical interactions. First, we performed co-immunoprecipitation of Gcn5-myc and Nse4-FLAG kleisin subunit to show the association between the SAGA and SMC5/6 complexes in vivo (Fig. 2A). The fission yeast cells carrying Nse4-FLAG (with or without Gcn5-myc) were lysed and proteins were precipitated with an anti-myc antibody. A small amount of Nse4-FLAG was specifically recovered in the Gcn5-myc precipitates but not in the control experiment without myc-tagged Gcn5, suggesting a weak or transient association between SAGA and SMC5/6 in the yeast cells.

Next, we used our panel of yeast two-hybrid (Y2H) SMC5/6 subunits to test their interactions with the HAT module (Ada2, Ada3, Gcn5 and Sgf29) subunits. Two of the HAT module subunits, Ada2 and Gcn5, bound the Nse3 subunit (Fig. 2B and Additional file 1: Fig. S2B). The fragment analysis showed that Gcn5 bound the N-terminal part of Nse3(aa1-190), whilst Ada2 interacted with its C-terminal WHB domain (Nse3(aa200-307); Fig. 2B and Additional file 1: Fig. S2C; [45]). In addition, Ada2 bound the N-terminal region of Nse2 and the coiled-coil arm of Smc5 [46]. Altogether, the Y2H data show multiple interactions between several SAGA and SMC5/6 subunits, and the co-immunoprecipitation experiment demonstrates the association between SAGA and SMC5/6 in vivo*.*

### SMC5/6 and SAGA are required for efficient DNA repair

**Fig. 3:**
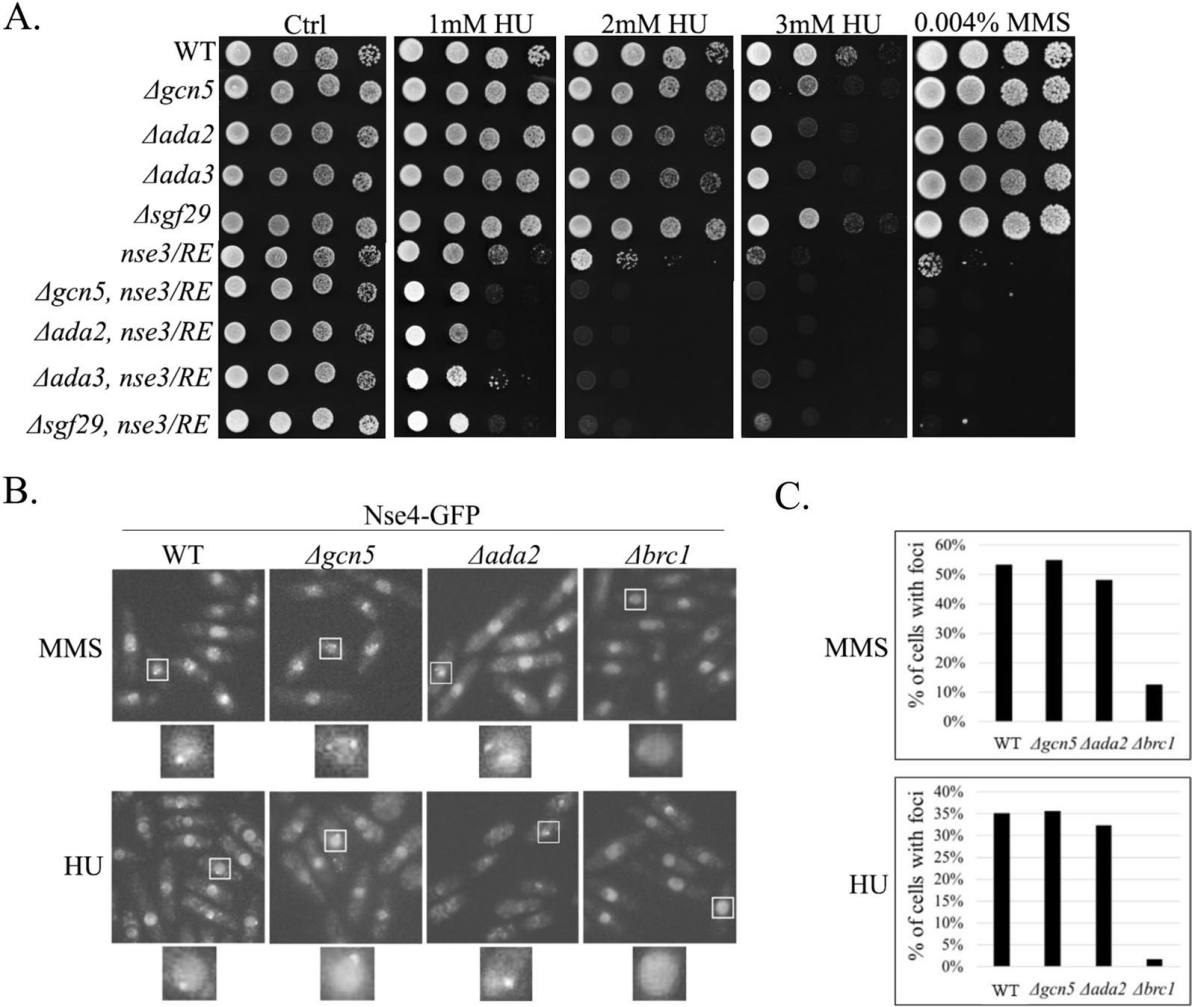
SMC5/6 and SAGA are required for efficient DNA repair. **A** Sensitivity of the SMC5/6 and SAGA mutants to genotoxins. Ten-fold serial dilutions of the yeast strains were plated onto YES media containing indicated concentrations of the hydroxyurea (HU) or methyl methane sulfonate (MMS). The double mutants were more sensitive than their respective single mutant counterparts, suggesting the non-redundant functions of SMC5/6 and SAGA in DNA repair. At least three independent drop tests were carried out, and only one of them is displayed as representative. **B** Live-cell microscopy of endogenous Nse4-GFP upon HU and MMS treatment, respectively. The Nse4-GFP foci were present in the WT and Δ*gcn5* cells but largely absent in Δ*brc1* cells. **C** Quantification of the data in **B** suggests that the localization of SMC5/6 to the DNA-damage foci is independent of SAGA

The SAGA complex plays an important role in transcription regulation and DNA repair by facilitating the accessibility of chromatin to transcription factors and repair proteins [43, 47–50]. Deletion mutants of most SAGA genes were sensitive to hydroxyurea (HU; [43]) and SAGA mutations increased the sensitivity of the *smc5/6* hypomorphic mutants to HU and other DNA-damaging agents (Fig. 3A, Additional file 1: Figs. S2A, and S3A). These data suggest either a direct role of the SAGA complex in facilitating SMC5/6 access to chromatin at sites of DNA damage or its indirect, independent role.

Upon DNA damage, SMC5/6 accumulates in foci in a Brc1-dependent way [51, 52]. To assess whether SMC5/6 chromatin accessibility at these DNA-damage sites is directly facilitated by SAGA, we analysed the Nse4-GFP foci in the Δ*gcn5* and Δ*ada2* mutants (Fig. 3B). We observed no difference between the frequency of cells with foci in the wild-type (WT), Δ*gcn5* and Δ*ada2* after treatment with either MMS (methyl methane sulfonate) or HU. In contrast, the number of foci in the Δ*brc1* mutant was strongly reduced (Fig. 3C). These results indicate no direct involvement of SAGA in SMC5/6 localization to sites of DNA damage. Instead, they suggest that the observed HU phenotype results from additive effects of the SAGA and SMC5/6 complexes during DNA-damage repair.

### SAGA targets SMC5/6 to gene regions

**Fig. 4:**
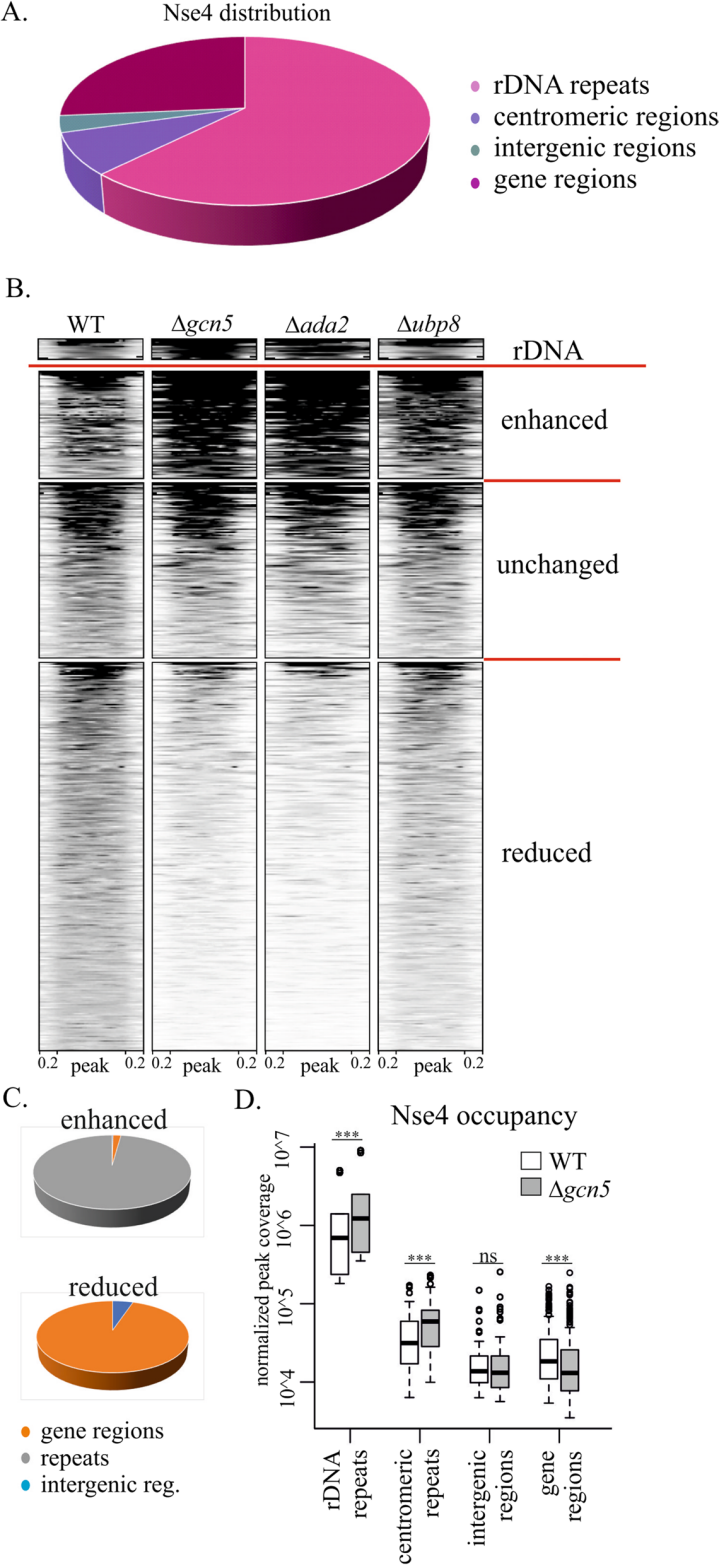
SMC5/6 distribution is dependent on the SAGA HAT module. **A** The pie chart shows the distribution of the Nse4-FLAG peak areas in the different genome regions in the WT fission yeast cells. Most SMC5/6 is localized to the repetitive regions (like rDNA and centromeres), with the highest occupancy of the rDNA repeats. A significant portion of the Nse4-FLAG is localized within the intergenic regions or genes. **B** The heatmap diagrams compare the occupancy of Nse4-FLAG peaks in the WT, Δ*gcn5,* Δ*ada2* and Δ*ubp8* mutant cells (as identified in the WT). The top part shows peaks enhanced in Δ*gcn5* and Δ*ada2* (enhanced)*,* whilst the bottom part clusters peaks reduced in the SAGA HAT module deficient cells (reduced). Peaks in the rDNA repeats are shown separately as these chromosome regions are not fully assembled and annotated in the *S. pombe* reference genome and exert a different range of coverage values (rDNA). The Nse4-FLAG peaks (normalized to median 760 bp width) and their surrounding regions (200 bp upstream and 200 bp downstream) are shown. **C** The pie charts show the distribution of the enhanced (top) and reduced (bottom) Nse4-FLAG peak areas in Δ*gcn5*. The SMC5/6 accumulation is mainly enhanced at the repetitive loci. The SMC5/6 localization is primarily reduced in gene regions. **D** The box plot graph compares the Nse4-FLAG occupancy in WT and Δ*gcn5* cells. The paired two-sided Wilcoxon statistical test was used: ns, non-significant; ***p < 0.001

In addition to the accessibility of chromatin to DNA repair and transcription factors, SAGA facilitates chromatin accessibility to condensin complexes [24]. Therefore, we determined the SMC5/6 localization in the unchallenged WT, Δ*gcn5*, Δ*ada2* and Δ*ubp8* cells using chromatin immunoprecipitation of Nse4-FLAG followed by deep sequencing (ChIP-seq). In the WT cells, most SMC5/6 localized to the repetitive regions (like rDNA, centromeres or tDNA copies), with the highest occupancy at the rDNA repeats (Fig. 4A) consistent with previous reports [53–58]. Interestingly, we identified 331 Nse4-FLAG peaks (representing ¼ of the total Nse4 occupancy; Fig. 4A) that localized to gene regions.

In Δ*gcn5* and Δ*ada2* mutants, SMC5/6 distribution was altered, whilst SMC5/6 occupancy in the Δ*ubp8* mutant was similar to that in WT cells. A heatmap clustering analysis showed that the Nse4-FLAG peaks were either enhanced, unchanged or reduced in the Δ*gcn5* and Δ*ada2* mutants (Fig. 4B). The peaks were mainly enhanced at the repetitive sequences (Fig. 4C), whilst most peaks in the intergenic regions were not changed (Fig. 4D). Strikingly, most peaks within the gene regions showed reduced Nse4 occupancy (252 out of 331; Fig. 4C and D), suggesting that the SAGA HAT module targets SMC5/6 to gene loci.

### SAGA plays a role in facilitating chromatin accessibility to SMC5/6 in specific regions

**Fig. 5:**
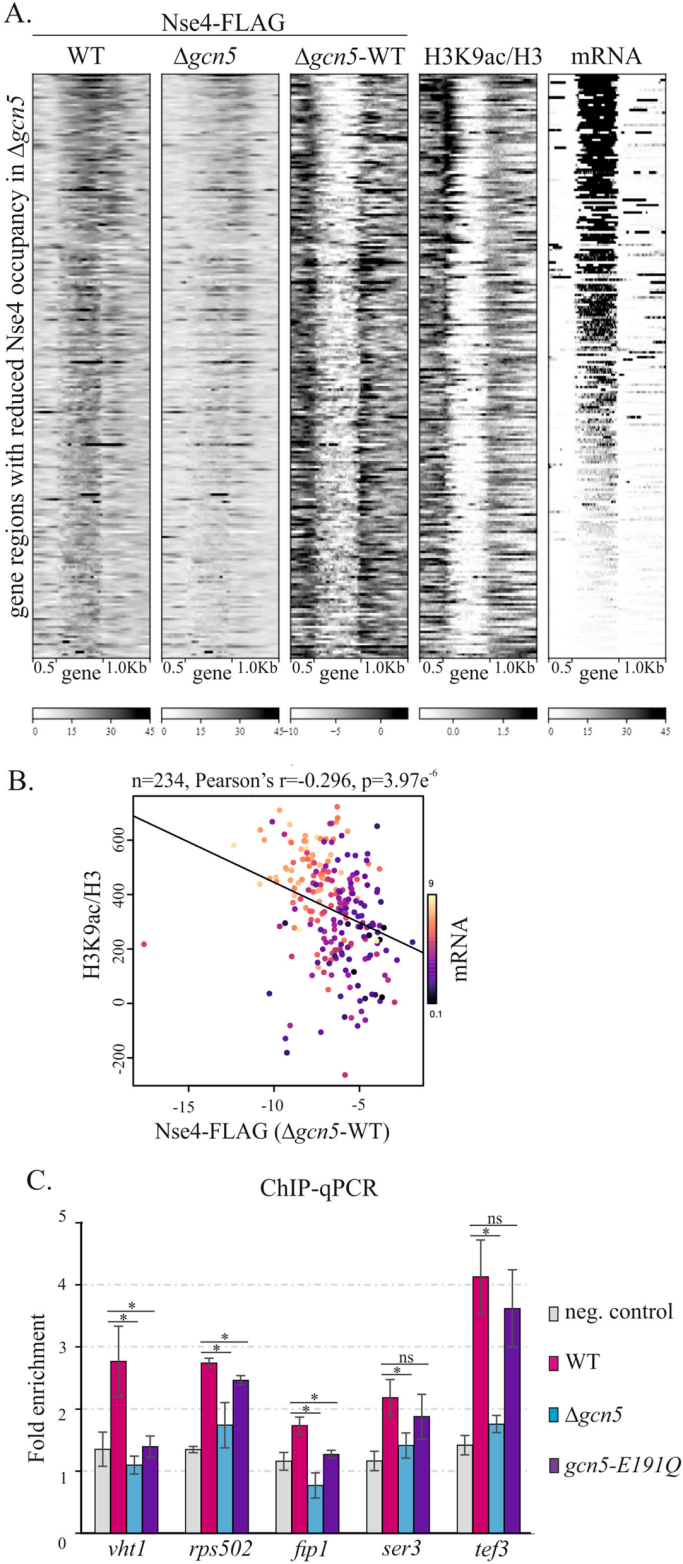
The SMC5/6 accumulation correlates with the H3K9 acetylation status. **A** Heatmap statistical analysis of the loci with reduced SMC5/6 occupancy upon the *gcn5* deletion. The Nse4-FLAG signals from WT and Δ*gcn5* are compared, and their differential plot is shown in the middle panel (Δ*gcn5-*WT). The results from ChIP-seq analysis of H3 and H3K9ac are shown (H3K9ac/H3). In addition, transcriptomic (RNA-seq) data are included (mRNA). The genes (normalized to 1 kb width) and their surrounding regions (500 bp upstream of TSS and 1 kb downstream of TTS) are shown. **B** Scatter plot analysis shows a strong correlation between the drop of SMC5/6 accumulation upon *gcn5* deletion (*X*-axis) and H3K9-acetylation status (*Y* axis) of gene regions (and their transcription levels; colour scale). **C** The results of chromatin immunoprecipitation followed by quantitative PCR (ChIP–qPCR) at selected gene loci are shown. Strains 503 (neg. control), MMP21 (WT, containing Nse4-FLAG), Nse4-FLAG Δ*gcn5* (Δ*gcn5*) and Nse4-FLAG *gcn5-E191Q (gcn5-E191Q*) were analysed. The fold enrichment was calculated against the negative *slx9* locus (mean ± standard deviation, *n* ≥ 3 biological replicates). The unpaired Wilcoxon statistical test was used: ns, non-significant; *p < 0.05

The SAGA HAT module acetylates histone H3 at its lysine K9 and K14 residues [59, 60]. To determine the H3K9ac distribution in our fission yeast cells, we performed ChIP-seq using an anti-H3K9ac antibody. A heatmap clustering analysis showed that the magnitude of decrease in SMC5/6 occupancy in gene bodies in the Δ*gcn5* mutant (Δ*gcn5-*WT plot; Fig. 5A and B) correlated with H3K9ac levels around the transcription start site and with transcript levels [61]. It suggests that the SAGA-dependent H3K9 acetylation may facilitate the accessibility of chromatin to SMC5/6 at gene regions.

To assess the role of the SAGA-dependent acetylation further, we used the *gcn5*-*E191Q* acetyltransferase-dead mutant [50]. First, we found that the *gcn5*-*E191Q ts* phenotype was exacerbated by *nse3-R254E,* similar to Δ*gcn5* (Additional file 1: Fig. S3D). Second, the *gcn5*-*E191Q nse3-R254E* double mutant was as sensitive to DNA-damaging agents as the Δ*gcn5 nse3-R254E* double mutant. Finally, using ChIP-qPCR analysis, we observed reduced Nse4-FLAG occupancy in the *gcn5*-*E191Q* mutant at selected gene regions similar to Δ*gcn5* (Fig. 5C), although some loci exhibited only a modest drop in SMC5/6 levels. Altogether, our results suggest an important role for the SAGA HAT module in targeting and facilitating the accessibility of chromatin to SMC5/6 at gene loci.

## Discussion

Here, we showed a new functional partnership between SAGA and SMC5/6 complexes. These complexes exhibited negative genetic interactions and physical associations mediated via multiple protein–protein interactions. *Gcn5* deletion reduced SMC5/6 occupancy specifically at the gene regions but did not affect the formation of SMC5/6 foci upon DNA damage.

It was recently shown that plant ADA2b binds SMC5 and assists in the localization of SMC5/6 to DNA-damage sites [62]. Later studies uncovered an ADA2b-diRNA pathway, involving specifically ADA2b without the other SAGA subunits, targeting SMC5/6 to DNA-damage foci in plants [63]. In contrast, our Y2H results show multiple interactions between several SAGA and SMC5/6 subunits (Fig. 2B). In addition, our co-immunoprecipitation experiment shows indirect interaction between Nse4-FLAG and Gcn5-myc subunits. As Nse4-FLAG and Gcn5-myc proteins incorporate into their respective complexes normally (Additional file 1: Fig. S4A; [39]), their co-immunoprecipitation confirms the association between SAGA and SMC5/6 complexes in vivo (Fig. 2A).

Furthermore, we and others showed that the SMC5/6 targeting to DNA-damage sites depends on the BRCT domain-containing Brc1 protein but not on Ada2 or Gcn5 (Fig. 3; [51]) in fission yeast, suggesting a plant-specific function of the ADA2b-SMC5 interaction in DNA-damage response. Future studies may show if SAGA likewise facilitates the accessibility of chromatin to SMC5/6 at gene loci in plants. Interestingly, we found an interaction between human hTADA2B and hNSE4a/b subunits (Additional file 1: Fig. S2D), suggesting that physical association between the SAGA and SMC5/6 complexes is conserved. It will be interesting to examine the functional relationships between human SAGA and SMC5/6 complexes, and explore the SAGA-SMC5/6 relationship further.

Consistent with the lack of effect of SAGA on SMC5/6 localization to sites of DNA damage, the SMC5/6 levels at repetitive regions (which are prone to DNA damage even without any genotoxic treatment) were not reduced in Δ*gcn5* and Δ*ada2* deletion mutants (Fig. 4). In our ChIP-seq experiments, the repetitive loci were instead enriched for SMC5/6 upon SAGA HAT deletion. As the SMC5/6 accumulation at repetitive regions depends on H3K9 methyltransferase Clr4 in fission yeast [53], we speculate that reduced acetylation levels promoted an increase in methylation levels upon *gcn5* acetyltransferase deletion, indirectly stimulating the SMC5/6 localization to repetitive heterochromatic regions [64].

Interestingly, enhanced SMC5/6 accumulation at repetitive regions could not rescue *smc5/6* phenotypes. Instead, *smc5/6 saga* double mutants exhibited additive growth defects, suggesting that either increased SMC5/6 accumulation at repetitive regions is toxic or reduced SMC5/6 loading at gene regions exacerbates *smc5/6* problems. Although we cannot exclude the former indirect effect, we found the latter possibility more straightforward. In this case, the reduced SMC5/6 targeting to gene regions upon *gcn5* deletion would exacerbate the DNA-binding defect of the *nse3-R254E* mutant, resulting in a more severe double mutant phenotype (Fig. 1).

**Fig. 6:**
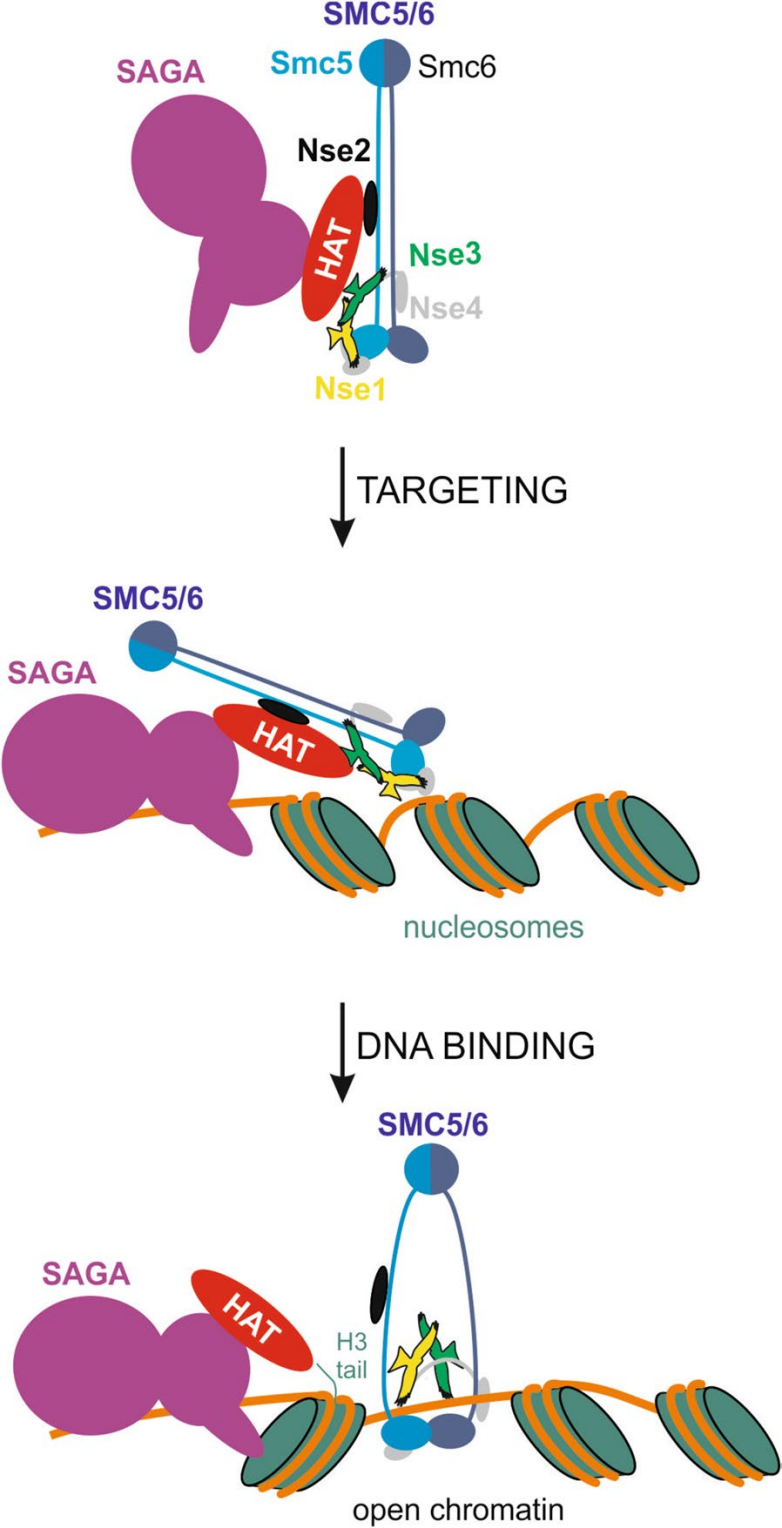
Hypothetical model of SAGA-mediated loading of SMC5/6 complex. The SAGA histone acetyltransferase (HAT; red) module binds several SMC5/6 subunits (top panel; Nse2: black, Smc5 arm: blue, and Nse3: green; model based on Ref. [15]). SAGA complex (violet; model based on Ref. [80]) binds promoter regions and targets rod-shaped SMC5/6 complex to specific sites (middle panel). Then, Gcn5-mediated acetylation of the H3 histone tails (dark green) helps to open chromatin (at least in a subset of cases) and expose free DNA to SMC5/6 (bottom panel). Open chromatin stimulates Nse3-mediated binding to free DNA [22] accompanied by SMC5/6 conformational change. Such ring-shaped conformation of SMC5/6 (model based on [17]) completely or partially dissociates from the SAGA complex (Additional file 1: Fig. S4)

Based on our data, we propose the following model for SAGA-dependent loading of SMC5/6: 1. SAGA binds SMC5/6 and targets it to specific gene regions; 2. H3K9 acetylation further helps open chromatin to allow SMC5/6 K-compartment physically bind DNA (Fig. 6). Interestingly, the SMC5/6 subunits (Nse2, Nse3 and Smc5 arm) are aligned at the surface of the rod-shaped complex for binding to the SAGA complex (Fig 2B and Additional file 1: Fig. S4B; [15]). In contrast, the Nse3 subunit is mostly buried inside the ring-shaped complex (Additional file 1: Fig. S4C; [17]), and its C-terminal WHB domain is directly bound to DNA. Therefore, we hypothesize that SAGA binds rod-shaped SMC5/6 first and brings it to its target gene region (Fig. 6, targeting step). At the target regions, SAGA acetylates H3 histones and facilitates further chromatin opening. This positions SMC5/6 to close proximity to free DNA and stimulates its direct binding to DNA. Nse3 binding to DNA results in the conformational change to the ring shape and complete or partial dissociation from the SAGA complex (Fig. 6, DNA-binding step).

This model implies that SMC5/6 loading is primarily dependent on the SMC5/6 binding to SAGA (Fig. 6, targeting step). The second DNA-binding step requires free DNA. This step depends on the open state of the chromatin, which might already be present at the target region (independent of SAGA) or induced by Gcn5-mediated histone acetylation. This two-step model explains different SMC5/6 levels in *gcn5* deletion and *gcn5-E191Q* mutant at different loci (Fig. 5C). Loading of SMC5/6 is already blocked in the first step in *gcn5* deletion, resulting in reduced SMC5/6 accumulation. In the acetyltransferase-dead *gcn5-E191Q* mutant, SAGA binds SMC5/6 and brings it to the target region in the first step, whilst the SMC5/6 binding to DNA depends on the chromatin state at particular loci. Open state of some loci may entirely depend on SAGA HAT activity, then SMC5/6 DNA binding is blocked in *gcn5-E191Q* (Fig. 6, middle panel)*,* similar to *gcn5* deletion (Fig. 5C, *vht1* locus). Other loci may provide free DNA sufficient for SMC5/6 loading even without Gcn5-assisted chromatin opening in *gcn5-E191Q* (loci with only modestly or partially reduced SMC5/6 levels in Fig. 5C, like *tef3* locus). Future studies will address the detailed mechanism of SMC5/6 loading.

## Conclusions

Here we described the new role of the SAGA complex in targeting SMC5/6 to specific gene regions, which is likely mediated via multiple direct interactions between subunits of these two complexes. In addition, SAGA may further assist in SMC5/6 chromatin loading through its acetyltransferase activity. Our new findings support previous views that different factors target SMC5/6 to different genomic regions [35, 52, 53, 65]. For example, Brc1 targets SMC5/6 to DNA-damage sites via its interaction with phosphorylated H2A (the equivalent of the targeting step in Fig. 6; [65]). Although it is not clear how SMC5/6 reaches free DNA in this case, nucleosome-free DNA structures are known to be available during DNA-damage repair. In comparison, SAGA targets SMC5/6 to unchallenged chromatin and can also assist in providing free DNA for SMC5/6 loading via chromatin acetylation. This SAGA-dependent loading likely constitutes a more general mechanism, as SAGA was also shown to assist in loading condensin in fission yeast [24]. In conclusion, our data outline the interplay between two key chromatin complexes, SMC5/6 and SAGA.

## Methods

### Yeast techniques

Standard fission yeast genetic techniques were used [66]. Yeast strains were crossed and sporulated either at 25 °C (*ts* mutants) or 28 °C (non-*ts* mutants). Tetrad analysis was carried out on Singer MSM300 (Singer, UK). The deletion integrations were verified on both ends by PCR with specific primers to the G418 cassette and genomic sequence of a deleted gene (approximately 600–800 bp from start or end). The PCR products were sequenced.

*Schizosaccharomyces pombe* cultures were grown to the mid-log phase, and serial tenfold dilutions were spotted onto rich media with the indicated dose of DNA-damaging agent (hydroxyurea or methyl methane sulfonate). Subsequently, plates were incubated at the indicated temperatures (25, 28 or 37 °C) for 3–4 days. At least three independent drop tests were carried out, whilst only one representative plate was displayed in the figure. Selective media were supplemented with Nourseothricin (cloNAT, 100 μg/ml, Jena Bioscience), G418 (100 μg/ml; Applichem) and/or cycloheximide (100 μg/ml; Sigma).

### Yeast genetic screens

The pAW8-Nse3 integration construct [22] was modified for use in the PEM2 strain as follows. The *Sph*I site was mutated to *Xho*I using a site-directed mutagenesis kit (Agilent Technologies; primers: LJ48 and LJ49; Additional file 1: Table S4). The cloNAT cassette was amplified (LJ42 and LJ43) and inserted into the *Xho*I site in front of the Nse3 gene using the In-fusion cloning kit (Takara). A 650 bp-long genomic sequence (upstream of Nse3; LJ44 and LJ45) was inserted in front of the cloNAT cassette (using *Xho*I) to ensure its proper integration into the *S. pombe* genome. The mutant cloNAT-*nse3-R254E* construct was created using site-directed mutagenesis (R254E_F and R254E_R primers; [22]). For the yeast transformation, the WT and mutant cloNAT constructs were cleaved by *Spe*I, and the 3246 bp long fragment was purified from agarose gel by Gel Extraction Kit (Qiagen). Approximately 1 μg of purified DNA was transformed into the PEM2 strain [31] by standard LiAc protocol. The proper integration of the cloNAT-Nse3 cassette and *rlp42* mutation was checked by PCR and sequencing. The *nse3*-*R254E* PEM2 strain phenotypes were compared with the original *nse3*-*R254E* strain (Additional file 1: Fig. S1A; [22]).

The WT and *nse3*-*R254E* mutant PEM2 strains (YLJ222 and YLJ228; Additional file 1: Table S5) were crossed with the *S. pombe* haploid deletion library (BIONEER, version 5, https://us.bioneer.com) according to the published protocol [31]. The screen was repeated twice using the Rotor HDA robot (Singer, UK). The plate images were taken by a Canon EOS Rebel T3i camera, and the individual colony size was measured. The viability of single deletion mutants (control WT plates) against double mutants (test *nse3*-*R254E* plates) was compared using SGAtools online platform [33]. Genes with a score less than − 0.25 were chosen as potential negative interactors (Additional file 1: Table S1).

The resulting group of 79 genes was analysed by the Gene ontology tool BiNGo [67], which is a plugin of the Cytoscape online platform [34]. The genes were classified according to the Pombase GO database into Biological processes and Cellular component categories, respectively [68]. The default parameters with a 0.05 significance level were applied for both categories.

### Yeast two-hybrid analysis

The Gal4-based Y2H system was used to analyse SMC5/6-SAGA interactions [69]. *S. pombe ada2*, *ada3*, *gcn5* and *sgf29* genes were PCR amplified from genomic DNA (primers used for *ada2* and *gcn5* cloning are listed in Additional file 1: Table S4). All inserts were cloned into respective sites of the pGBKT7 or pGADT7 vectors using the In-Fusion cloning system. pGBKT7-Nse2(aa2-178) was described in [9]. pGADT7-Nse3(aa1-190) was prepared by mutagenesis of 191^st^ aa to STOP codon in pGADT7-Nse3(aa1-328) [70]. The Nse3(aa200-307) fragment was cut out from pTriEx4-Nse3(aa200-307) [45] by *Nco*I-*Xho*I enzymes and cloned into pGADT7. The Smc5(aa170-225 + 837-910) fragment was amplified from the Smc5(aa2-225 + 837-1065) construct [71] and inserted into the *Nco*I-*Not*I sites of pGBKT7.

The pairs of pGBKT7 and pGADT7 constructs were co-transformed into the *Saccharomyces cerevisiae* PJ69–4a strain by standard LiAc transformation protocol and selected on SD-Leu, -Trp plates. Drop tests were carried out on SD-Leu, -Trp, -His (with 0.3, 0.5, 1, 3, 5 or 10 mM 3-aminotriazole) plates at 28 °C. Each combination of partners was co-transformed and tested at least twice.

### Co-immunoprecipitation of *S. pombe* proteins

Logarithmically growing YLJ507 and MMP21 cells (Additional file 1: Table S5, Fig. S3B) were cultivated in a rich medium at 28 °C (OD_595_ = 0.4–0.7). 5 × 10^8^ cells were harvested by centrifugation (3 min, 4 °C, 5000 rpm) and washed with 10 ml of ice-cold PBS. Pellets were stored in the 2 ml screw cup tubes at − 80 °C. The crude yeast extracts were prepared in 400 μl CHIP lysis buffer (50 mM HEPES, pH 7.5, 140 mM NaCl, 1 mM EDTA, 1% Triton X-100, Complete EDTA-free protease inhibitor cocktail tablets, Roche) with half volume of glass beads (Sigma) in 2 ml low binding tubes using FASTprep-24 (MP Biomedicals; 5 times, 30 s, 6.5 speed). The suspension was recovered by piercing the bottom of the tube with a needle, placing it into a new 2 ml tube, and centrifugation (3 min, 4 °C, 5000 rpm). The beads were washed with 200 μl of CHIP lysis buffer (3 min, 4 °C, 5000 rpm). The collected suspensions were clarified by centrifugation (15 min, 4 °C, 15,000 rpm), and the supernatant was transferred to new low-binding tubes. 40 μl of cell extract was taken for input control. Immunoprecipitation was carried out by adding 2 μl mouse anti-myc antibody (2276S, Cell Signalling) to the cell extracts and incubation for 2 h at 4 °C. 20 μl of protein G-coated Dynabeads (Invitrogen) were washed twice with 1 ml CHIP lysis buffer and resuspended in 60 μl of CHIP lysis buffer, then added to the extract with anti-myc antibody and incubated overnight at 4 °C. The beads were pelleted using a magnetic rack, and the unbound fraction (40 μl) was taken. Beads were washed four times with 1 ml CHIP lysis buffer, and proteins were eluted by 40 μl of 1× SDS loading buffer. After 15 min incubation at room temperature, the supernatant (bound fraction) was recovered. All fractions were analysed by western blotting using mouse anti-myc-HRP (R951-25, Thermo Fisher) and mouse anti-FLAG-HRP (F1804-1MG, Sigma) antibodies, respectively.

### Protein modelling

The AlphaFold tools [72, 73] were used to generate in silico fission yeast SMC5/6 subunits and complex models. Structural models were analysed as previously described [6, 74]. Structures were visualized using PyMOL Molecular Graphics System.

### Chromatin-immunoprecipitation analysis (ChIP)

#### Nse4-FLAG ChIP-seq

All strains (Additional file 1: Table S5) were cultivated into the mid-log phase (OD = 0.4–0.6) and incubated with 1% formaldehyde for 15 min at room temperature to cross-link DNA–protein complexes. Glycine was added to a final concentration of 125 mM, and the incubation continued for 5 min. 5 × 10^8^ cells were harvested and washed with 10 ml of ice-cold PBS. The yeast cell wall breakage was performed in 400 μl CHIP lysis buffer with half the volume of glass beads in 2 ml low-binding tubes using FASTprep-24. The suspension was washed two times with CHIP lysis buffer (15 min, 4 °C, 15,000 rpm), and 300 μl of the extract was sonicated with Bioruptor (Diagenode, 30 s ON/30 s OFF, High Power, 25 times) and clarified by centrifugation (15 min, 4 °C, 15,000 rpm), resulting in an average DNA fragment size of 300–500 bp. 5 μl of the sonicated precleared extract was taken as an input control sample.

Monoclonal anti-FLAG M2 antibody (F1804, Sigma) was diluted 1:150, incubated with precleared cell extract in 1.5 ml low-binding tube for 2 h on ice and precipitated overnight with Dynabeads protein G (Invitrogen). Precipitates were washed with 1 ml of CHIP lysis buffer, 1 ml of High Salt buffer (CHIP lysis buffer with 500 mM NaCl), 1 ml of Wash buffer (10 mM Tris–HCl at pH 8.0, 0.25 M LiCl, 0.5% NP-40, 1 mM EDTA) and 1 ml of TE buffer (20 mM Tris–HCl at pH 8.0, 1 mM EDTA). After elution (50 mM Tris at pH 8, 0.1% SDS, 10 mM EDTA) and de-crosslinking overnight at 65 °C, the DNA was purified using QIAquick PCR Purification Kit (Qiagen).

For ChIP-seq analysis, the input DNA samples were tested for DNA fragmentation and determination of DNA concentration by the Fragment analyser (Agilent). Input and immunoprecipitation (IP) samples with the best fragmentation and high concentration were used for the creation of NGS libraries (NEBNEXT ULTRA II DNA Library Prep kit, NEB) and sequencing (Illumina Next seq 500, Illumina).

#### H3K9ac/H3 ChIP-seq

Two independent replicates were performed. Cells were grown to the exponential phase (OD_600_ = 0.5) in the complex YES medium and fixed by adding formaldehyde to the final concentration of 1%. After 30 min incubation, the remaining formaldehyde was quenched by 125 mM glycine. Cells were washed with PBS and broken with glass beads. Extracted chromatin was sheared with the Bioruptor sonicator (Diagenode) using 15 or 30 cycles (for biological replicate 1 and 2, respectively) of 30 s ON/30 s OFF at high power settings. For all immunoprecipitations (IP) within a biological replicate, the same amount of chromatin extract was used (2.5 or 3.7 mg of total protein); 1/10 of the total chromatin extract amount was kept for input DNA control. For each IP, 5 μg of antibody (H3: Ab1791, H3K9ac: Ab4441, all Abcam) were incubated with the chromatin extract for 1 h at 4 °C with rotation. Then, 50 μl of BSA-blocked Protein A-coated magnetic beads (10002D, ThermoFisherScientific) were added to the chromatin extract-antibody suspension and incubated for additional 4 h at 4 °C with rotation. The precipitated material and input chromatin extract were de-crosslinked and treated with RNase A and proteinase K. DNA was purified using phenol–chloroform extraction and sodium acetate/ethanol precipitation. In biological replicate 2, DNA purification on AMPure XP beads (AC63880, Beckman Coulter) was performed after the phenol–chloroform extraction to remove low-molecular fragments and RNA. DNA concentration was measured using the Quantus fluorometer (Promega) and fragment size distribution was checked on Agilent Bioanalyser using the High Sensitivity DNA Assay. Library construction and sequencing (50 nt SE) were performed by BGI Tech Solutions (Hong Kong) using the BGISEQ-500 sequencing system.

#### NGS data analysis

The reference fission yeast *S. pombe* genome (2018-09-04) and annotation (2019-11-15) were downloaded from PomBase (https://www.pombase.org/; [68, 75]). Read quality was checked using FastQC version 0.11.8 (https://www.bioinformatics.babraham.ac.uk/projects/fastqc/), and reads were aligned to the *S. pombe* genome using HISAT2 2.1.0 [76] and SAMtools 1.9 [77, 78]. Read coverage tracks (i.e. target protein occupancy) were then computed and normalized to the respective mapped library sizes using deepTools 3.5.1 [79]. The raw ChIP-seq data are available from the ArrayExpress (https://www.ebi.ac.uk/) database under the accession numbers E-MTAB-11081 and E-MTAB-12401.

WT fission yeast RNA-seq data were obtained from the NCBI Sequence Read Archive (https://www.ncbi.nlm.nih.gov/sra; datasets SRR8742773-SRR8742775; [61]). Reads were processed and analysed with the same tools as above. All relevant scripts for (ChIP-seq and RNA-seq) data processing and analysis are available from https://github.com/mprevorovsky/Palecek-Nse-SAGA.

#### ChIP-qPCR

The Nse4-FLAG strains (crossed with the Δ*gcn5* or *gcn5-E191Q* strain; Additional file 1: Table S5) were used. The untagged wild-type strain was used as a negative control. All cells were cultivated into the mid-log phase. Cells were then incubated with 1% formaldehyde for 10 min at room temperature to cross-link DNA–protein complexes. Chromatin immunoprecipitation was performed using a protocol described above for H3K9ac ChIP with the following modifications. Monoclonal anti-FLAG M2 antibody (F1804; Sigma) was diluted at 1:350 (5 µg/sample), incubated with 2 mg of total cell extract for 2 h at 4 °C with rotation and precipitated with Dynabeads protein G (Invitrogen). After overnight incubation, several washes, elution and de-crosslinking, the DNA was purified using phenol/chloroform method.

The relative amount of PCR product was quantified by qPCR using SensiFASTTM SYBR^®^ Hi -ROX Kit (Bioline). The sequences of primers used for the quantitative detection of the chromosomal loci are listed in Additional file 1: Table S6. Input DNA recovery was calculated as 2 squared [CT(input) − CT(immunoprecipitate)] and normalized to a negative locus *slx9*. Melt curve analysis was performed for each sample after PCR amplification to ensure that a single product was obtained.

### Microscopy

For the Nse4-GFP foci number determination, cells were grown in YES medium overnight, diluted to OD = 0.4 in the morning and treated with 0.03% MMS or 20 mM HU for 5 h at 30 °C. 2.5 μl of cell culture was mounted on the slides and GFP fluorescence was observed. Pictures were taken on the Axio Imager Z1 microscope, using a Plan-Apochromat 63× oil objective, the Axiocam CCD camera and processed with the AxioVision software (all by Zeiss). A minimum of 500 cells were counted in three independent experiments. For statistical evaluation, *p*-values were calculated using the *χ*^2^ test.

## Supplementary Information

**Additional file 1. **Additional methods, additional figures S1–S4, additional tables S1–S6.

## Data Availability

All relevant data are within the manuscript and its Additional files.
