# Supplementary material for: The SAGA histone acetyltransferase module targets SMC5/6 to specific genes
Source: Epigenetics Chromatin. 2023 Feb 16;16:6. doi: 10.1186/s13072-023-00480-z (PMC9933293; doi:10.1186/s13072-023-00480-z)
Supplement: Supplementary file 1 — Additional file 1. Additional methods, additional figures S1–S4, additional tables S1–S6. [file 13072_2023_480_MOESM1_ESM.pdf]

The SAGA histone acetyltransferase module targets SMC5/6 to specific genes

Mahrik L. [1,2], Stefanovie B. [1,2], Maresova A. [3], Princova J. [3], Kolesar P. [1], Lelkes E. [1], Faux C. [4], Helmlinger D. [4], Prevorovsky M. [3*], Palecek J.J. [1,2*]

[1] National Centre for Biomolecular Research, Faculty of Science, Masaryk University, Kotlarska 2, 61137 Brno, Czech Republic

[2] Mendel Centre for Plant Genomics and Proteomics, Central European Institute of Technology, Masaryk University, Kamenice 5, 62500 Brno, Czech Republic

[3] Department of Cell Biology, Faculty of Science, Charles University, Vinicna 7, 12800 Prague, Czech Republic

[4] Centre de Recherche en Biologie cellulaire de Montpellier, University of Montpellier, CNRS, 1919 route de Mende, 34293 Montpellier Cedex 05, France

Additional file 1

Materials and methods

Yeast two-hybrid analysis

Most of the *S. pombe* and human SMC5/6 Y2H plasmids were prepared previously: pGADT7-spNse1(aa1-232), pGBKT7-spNse1(aa1-232), pACT2-spNse2(aa1-250), pGBKT7-spNse2(aa1-250), pGADT7-spNse3(aa1-328), pGBKT7-spNse3(aa1-328), and pGBKT7-spNse4(aa1-300) in [1]; pACT2-spNse4(aa1-300), pGADT7-hNSE1(aa1-266), pGADT7-hNSE3(aa55-292) and pGADT7-hNSE4b(aa1-333) in [2]; pACT2-spSmc5(aa1-1065), pGBKT7-spSmc5(aa1-1065), pGBKT7-Smc5(aa2-323+732-1065) in [3]; pGADT7-spSmc6(aa1-1140) in [4]; pGADT7-hNSE2(aa1-247), pGADT7-hSMC5(aa4-1101), pGADT7-hSMC6(aa1-1091) in [5]. The TAFA2B cDNA was obtained from the human ORFeome collection (v8.1, http://horfdb.dfci.harvard.edu/hv5/hv8/index.php?page=home) and subcloned into the pACT2 vector in a single step using the Gateway technology. ORF was PCR

amplified using primers listed in Suppl. Table ST4 and cloned with In-Fusion cloning system to pGBKT7.

The spNse3(aa1-90) fragment was created by mutating 91st aa to STOP codon (Suppl. Table ST4) in pGADT7-spNse3(aa1-328) [1] using QuickChange Lightning Site-directed mutagenesis kit (Agilent Technologies). The spNse3(aa80-210) and spNse3(aa80-307) fragments were cleaved out by *Nco*I-*Xho*I from respective pTriEx-Nse3 plasmids [2] and inserted into the pGADT7 vector using T4 ligase protocol (New England Biolabs). hNSE4a(aa1-385) was cloned into the pGADT7 vector through *Nde*I-*Xho*I sites by the In-fusion cloning system (Takara). The spSmc5(aa600-835) fragment was amplified by JP295+JP259 primers and inserted into the *Nde*I-*Xho*I sites of pGBKT7 using the T4 ligase protocol.

SAGA purification

Fission yeast extracts were prepared, and purification of the SAGA complexes using the Spt7-TAP tag was performed as in [6]. Purified proteins were run on SDS-PAGE gel and stained with silver dye.

The other methods are described in the main text.

References

1. Sergeant J, Taylor E, Palecek J, Fousteri M, Andrews E, Sweeney S, et al. Composition and architecture of the Schizosaccharomyces pombe Rad18 (Smc5-6) complex. Mol Cell Biol. 2005;25(1):172-84. doi: 10.1128/MCB.25.1.172-184.2005. PubMed PMID: WOS:000226236900015.

2. Hudson JJR, Bednarova K, Kozakova L, Liao CY, Guerineau M, Colnaghi R, et al. Interactions between the Nse3 and Nse4 Components of the SMC5-6 Complex Identify

Evolutionarily Conserved Interactions between MAGE and EID Families. Plos One. 2011;6(2):14. doi: 10.1371/journal.pone.0017270. PubMed PMID: WOS:000287764100039.

3. Palecek J, Vidot S, Feng M, Doherty AJ, Lehmann AR. The SMC5-6 DNA repair complex: Bridging of the SMC5-6 heads by the Kleisin, NSE4, and non-Kleisin subunits. J Biol Chem. 2006;281:36952-9. PubMed PMID: 17005570.

4. Zabrady K, Adamus M, Vondrova L, Liao C, Skoupilova H, Novakova M, et al. Chromatin association of the SMC5/6 complex is dependent on binding of its NSE3 subunit to DNA. Nucleic Acids Research. 2016;44(3):1064-79. doi: 10.1093/nar/gkv1021. PubMed PMID: WOS:000371268700017.

5. Adamus M, Lelkes E, Potesil D, Ganji SR, Kolesar P, Zabrady K, et al. Molecular Insights into the Architecture of the Human SMC5/6 Complex. Journal of Molecular Biology. 2020;432(13):3820-37. doi: 10.1016/j.jmb.2020.04.024. PubMed PMID: WOS:000541931500007.

6. Laboucarié T, Detilleux D, Rodriguez-Mias RA, Faux C, Romeo Y, Franz-Wachtel M, et al. TORC1 and TORC2 converge to regulate the SAGA co-activator in response to nutrient availability. EMBO Rep. 2017;18(12):2197-218. Epub 20171027. doi: 10.15252/embr.201744942. PubMed PMID: 29079657; PubMed Central PMCID: PMCPMC5709762.

7. Hallett ST, Campbell Harry I, Schellenberger P, Zhou L, Cronin NB, Baxter J, et al. Cryo-EM structure of the Smc5/6 holo-complex. Nucleic Acids Res. 2022. Epub 20220822. doi: 10.1093/nar/gkac692. PubMed PMID: 35993814; PubMed Central PMCID: PMCPMC9458440.

8. Yu Y, Li S, Ser Z, Kuang H, Than T, Guan D, et al. Cryo-EM structure of DNA-bound Smc5/6 reveals DNA clamping enabled by multi-subunit conformational changes. Proc Natl

Acad Sci U S A. 2022;119(23):e2202799119. Epub 20220601. doi: 10.1073/pnas.2202799119. PubMed PMID: 35648833.

Figure legends

Figure S1. SMC5/6 genetic interactions

(**A**) Sensitivity of the original *nse3-R254E* (*nse3/RE*) cells and cells with the cloNAT-*nse3-R254E* construct integrated into the PEM2 background (NAT-*nse3/RE*). Ten-fold serial dilutions of these strains were plated onto YES media containing indicated concentrations of the hydroxyurea (HU) or methyl methane sulfonate (MMS). At least three independent drop tests were carried out, and only one of them was displayed as representative. (**B** and **D**) Tetrad analysis of the diploid fission yeast strains. The original *nse3-R254E* mutant strain was crossed with the indicated mutants and sporulated. Representatives of the tetrad analysis are shown with the double mutants indicated by triangles. SAGA subunits are colour-coded as in panel C. NuA4 subunits are in bold. (**C**) Schematic representation of the SAGA complex with differently coloured subunit modules. Blue, HAT module; orange, DUB module.

Figure S2. Genetic and physical interactions between the SMC5/6 and HAT module

(**A**) Phenotypes of the SMC5/6 and SAGA mutants. Ten-fold serial dilutions of the indicated strains were plated onto YES media and grown at 25°C (control) or 37°C. The *nse3-R*254E, *smc6*-74, and *smc6*-X mutations enhanced the sensitivity of the Δ*gcn5* mutant to the higher temperature (*ts* phenotype). In contrast, *nse2-SA* did not affect the *ts* phenotype of Δ*gcn5*. Nevertheless, all *smc5/6* mutants enhanced the sensitivity of Δ*gcn5* to the DNA damaging agents (HU or MMS). (**B - D**) The yeast two-hybrid (Y2H) system was used to determine protein-protein interactions between SMC5/6 and SAGA HAT module (Ada2 and Gcn5) subunits. The protein-protein interactions were scored by the growth of yeast PJ69 transformants on the plates without Leu, Trp, and His, containing 3-Amino-1,2,4-triazole. (**B**)

The full-length Gal4BD-Gcn5 and Gal4BD-Ada2 (left part) or Gal4AD-Ada2 (right part) hybrid constructs were co-transformed together with the Gal4AD (left panel) or Gal4BD (right panel) full-length SMC5/6 hybrid constructs. The Gal4AD-Nse3 interacted with both Gal4BD-Ada2 and Gal4BD-Gcn5; Gal4BD-Nse2 interacted with Gal4AD-Ada2. (**C**) Detailed Y2H analysis of the above (panel B) interactions and SMC5-Ada2 interaction. Gcn5 interacted with the N-terminal part of Nse3, and Ada2 bound its C-terminal WHB domain. Ada2 bound the N-terminal part of Nse2 and to the arm of SMC5 next to its Nse2-binding interface. Note that the Gal4BD-SMC5 CC arm and Gal4BD-Nse2 full-length constructs self-activated. Therefore, their interactions were scored at higher amino-triazole concentrations. In control experiments, empty pGADT7 and pGBKT7 vectors were used as indicated. (**D**) Human SMC5/6 subunits were tested for their binding to hTADA2B in Y2H. The full-length and hNSE3(aa55-292) Gal4AD hybrid constructs were used. Only Gal4AD-hNSE4a and Gal4AD-hNSE4b construct bound Gal4BD-hTADA2B. At least three independent drop tests were carried out, and only one of them was displayed as representative.

Figure S3. Sensitivity of the SMC5/6 and SAGA strains to genotoxins.

(**A** and **D**) Ten-fold serial dilutions of the indicated strains were plated onto YES media containing indicated concentrations of HU or MMS. The double mutants were more sensitive than their single mutant counterparts suggesting the non-redundant functions of SMC5/6 and SAGA in DNA repair. (**B**, **C**, and **E**) Comparison of the Nse4-FLAG strains with their parental counterparts. Ten-fold serial dilutions of the Nse4-FLAG strains and their parental counterparts were plated onto YES media with or without indicated concentrations of HU or MMS. The plates were grown at 25°C (or 37°C, if indicated). At least three independent drop tests were carried out, and only one of them was displayed as representative.

Figure S4. SAGA complex purification and positions of SMC5/6 subunits within different conformations.

(**A**) Purification of the SAGA complex using the Spt7-TAP tag from fission yeast extracts containing (+, DH815) or missing (-, DH783) Gcn5-myc. Purified proteins were run on SDS-PAGE gel and silver-stained. (**B**) The Nse2 (black), Smc5 arm (blue), and Nse3 (green) subunits are aligned at the surface of the rod-shaped SMC5/6 complex (model based on 7QCD structure from [7]). This conformation is well-spaced to bind the SAGA subunits Ada2 (red) and Gcn5 (dark red). The Nse3 WHA and WHB domains are accessible for binding to both Ada2 and Gcn5 (left enlarged view). (**C**) In contrast, the Nse3 is mostly buried inside the ATP-bound ring-shaped complex (model based on both 7QCD and 7TVE structures from [7, 8]) and insulated from Smc5 by Nse1 (yellow). In addition, its WHB domain is directly bound to DNA (orange) within the K-compartment (right enlarged view), suggesting that Nse3 could not bind Ada2 and Gcn5.

Tables

Table S1 - List of genes with significant SGA scores.

Table S2 - Summary of significantly enriched GO categories for Biological processes

Table S3 - Summary of significantly enriched GO categories for Cellular components

Table S4 - List of cloning primers

Table S5 - List of yeast strains

Table S6 - List of qPCR primers

Figure S1

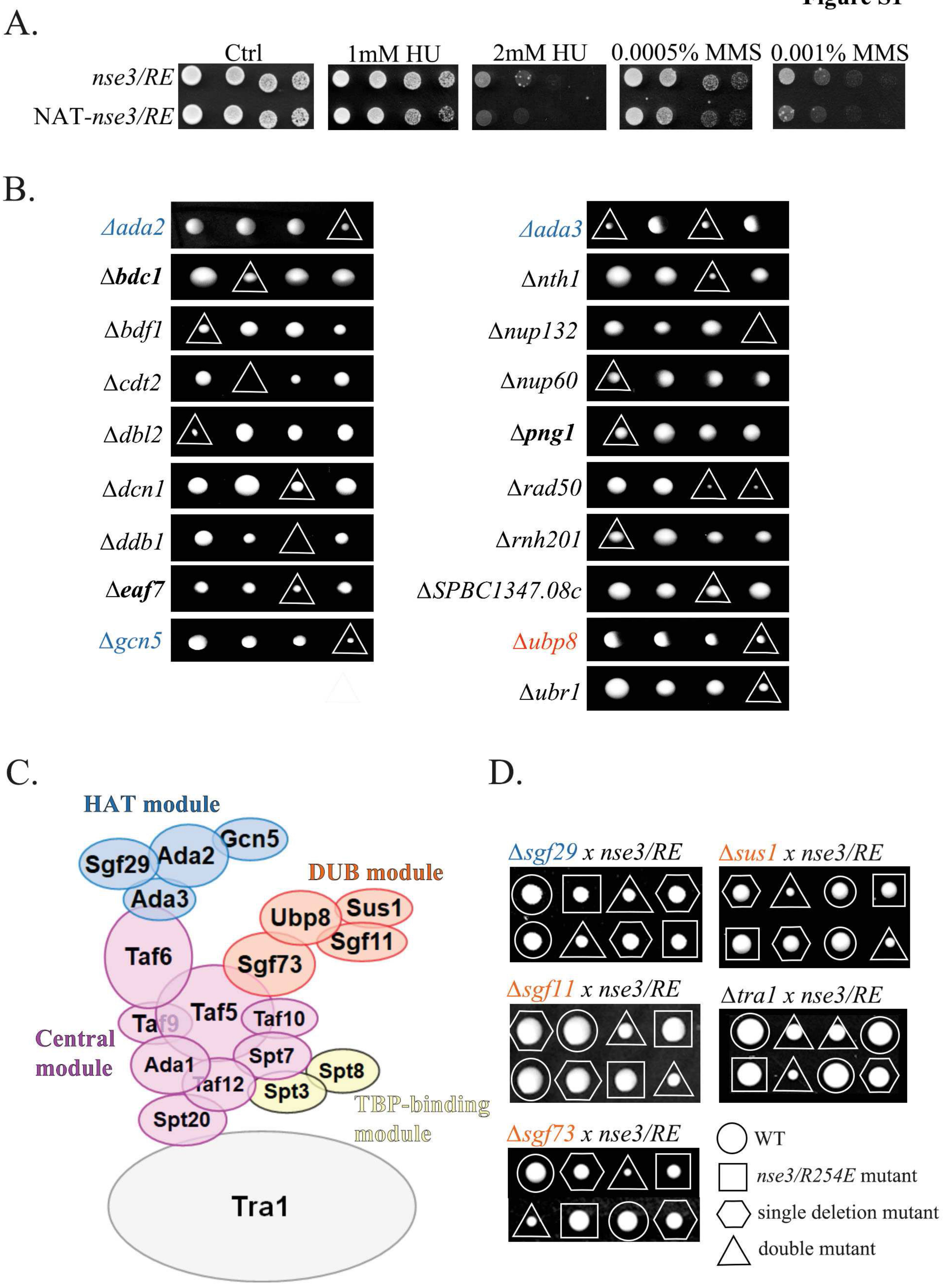

A.
Ctrl
1mM HU
2mM HU
0.0005% MMS
0.001% MMS
nse3/RE
NAT-nse3/RE
B.
Δada2
Δbdc1
Δbdf1
Δcdt2
Δdbl2
Δdcn1
Δddb1
Δeaf7
Δgcn5
Δada3
Δnth1
Δnup132
Δnup60
Δpng1
Δrad50
Δrnh201
ΔSPBC1347.08c
Δubp8
Δubr1
C.
HAT module
Gcn5
Sgf29
Ada2
Ada3
DUB module
Ubp8
Sus1
Sgf11
Sgf73
Taf6
Taf9
Taf5
Taf10
Central module
Ada1
Spt7
Spt8
Taf12
Spt3
TBP-binding module
Spt20
Tra1
D.
Δsgf29 x nse3/RE
Δsus1 x nse3/RE
Δsgf11 x nse3/RE
Δtra1 x nse3/RE
Δsgf73 x nse3/RE
WT
nse3/R254E mutant
single deletion mutant
double mutant

Figure S2

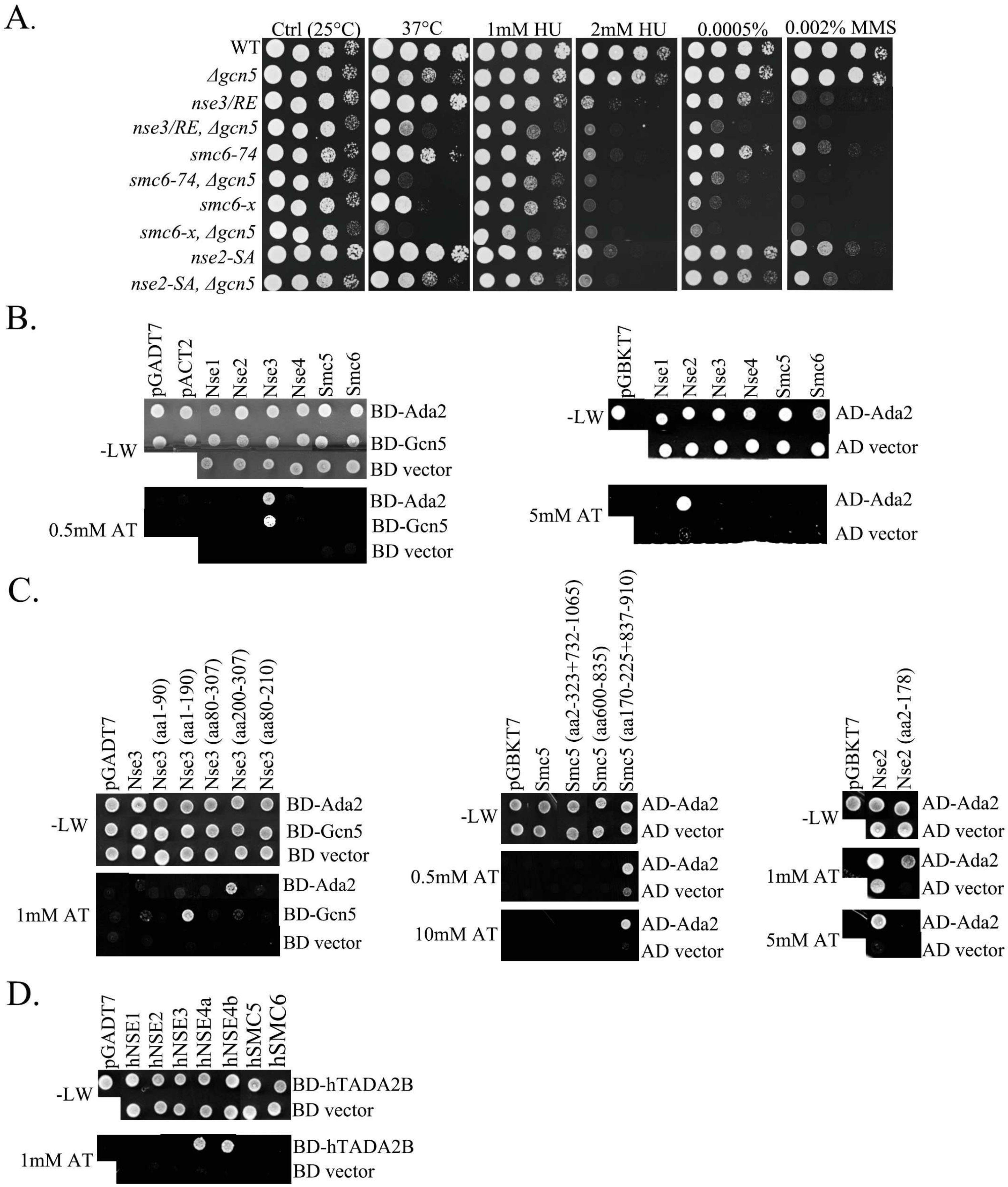
A.
Ctrl (25°C)
37°C
1mM HU
2mM HU
0.0005%
0.002% MMS
WT
Δgcn5
nse3/RE
nse3/RE, Δgcn5
smc6-74
smc6-74, Δgcn5
smc6-x
smc6-x, Δgcn5
nse2-SA
nse2-SA, Δgcn5
B.
pGADT7
pACT2
Nse1
Nse2
Nse3
Nse4
Smc5
Smc6
-LW
BD-Ada2
BD-Gcn5
BD vector
0.5mM AT
BD-Ada2
BD-Gcn5
BD vector
pGBKT7
Nse1
Nse2
Nse3
Nse4
Smc5
Smc6
-LW
AD-Ada2
AD vector
5mM AT
AD-Ada2
AD vector
C.
pGADT7
Nse3
Nse3 (aa1-90)
Nse3 (aa1-190)
Nse3 (aa80-307)
Nse3 (aa200-307)
Nse3 (aa80-210)
-LW
BD-Ada2
BD-Gcn5
BD vector
1mM AT
BD-Ada2
BD-Gcn5
BD vector
pGBKT7
Smc5
Smc5 (aa2-323+732-1065)
Smc5 (aa600-835)
Smc5 (aa170-225+837-910)
-LW
AD-Ada2
AD vector
0.5mM AT
AD-Ada2
AD vector
10mM AT
AD-Ada2
AD vector
pGBKT7
Nse2
Nse2 (aa2-178)
-LW
AD-Ada2
AD vector
1mM AT
AD-Ada2
AD vector
5mM AT
AD-Ada2
AD vector
D.
pGADT7
hNSE1
hNSE2
hNSE3
hNSE4a
hNSE4b
hSMC5
hSMC6
-LW
BD-hTADA2B
BD vector
1mM AT
BD-hTADA2B
BD vector

Figure S3

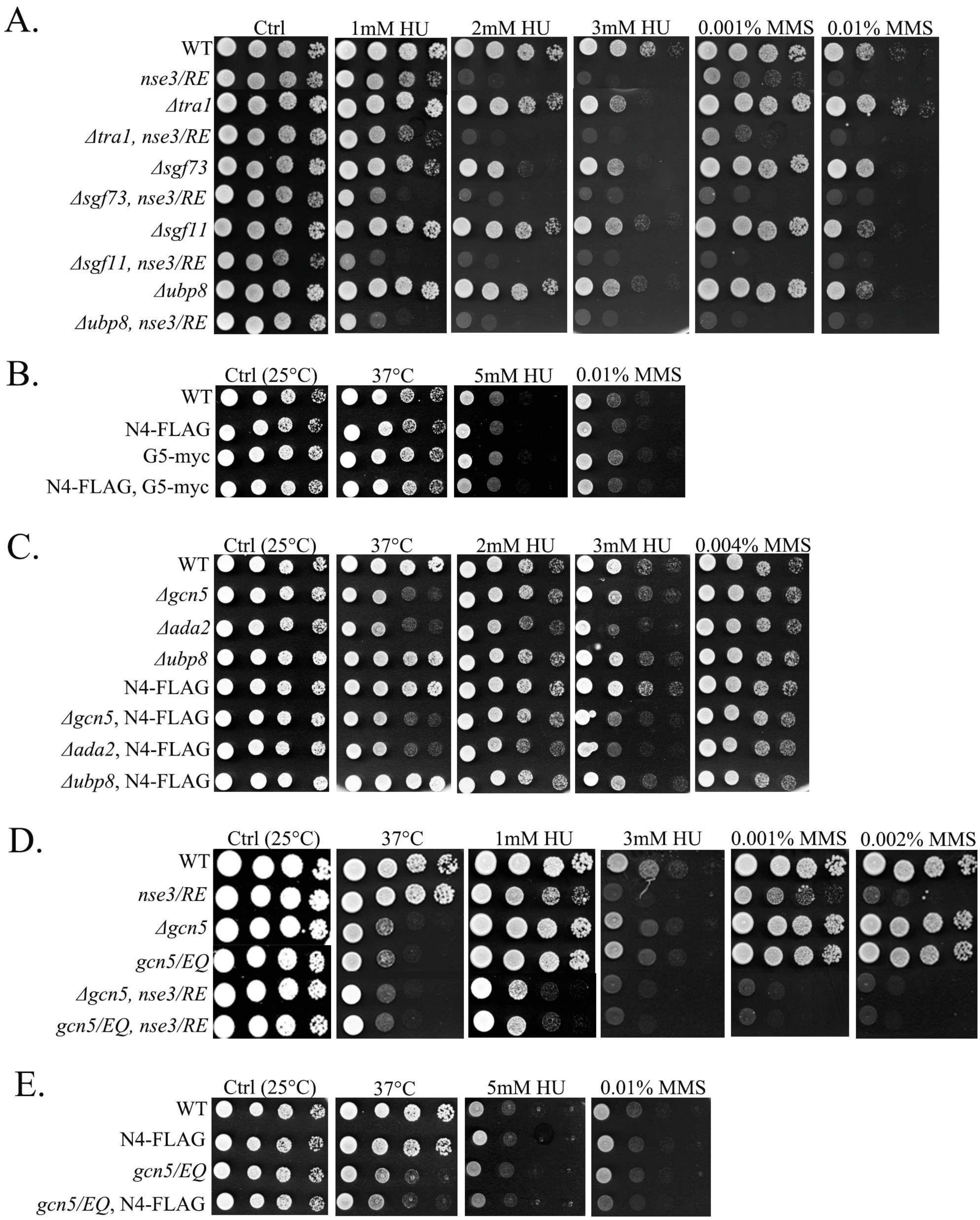
A.
Ctrl
1mM HU
2mM HU
3mM HU
0.001% MMS
0.01% MMS
WT
nse3/RE
Δtra1
Δtra1, nse3/RE
Δsgf73
Δsgf73, nse3/RE
Δsgf11
Δsgf11, nse3/RE
Δubp8
Δubp8, nse3/RE
B.
Ctrl (25°C)
37°C
5mM HU
0.01% MMS
WT
N4-FLAG
G5-myc
N4-FLAG, G5-myc
C.
Ctrl (25°C)
37°C
2mM HU
3mM HU
0.004% MMS
WT
Δgcn5
Δada2
Δubp8
N4-FLAG
Δgcn5, N4-FLAG
Δada2, N4-FLAG
Δubp8, N4-FLAG
D.
Ctrl (25°C)
37°C
1mM HU
3mM HU
0.001% MMS
0.002% MMS
WT
nse3/RE
Δgcn5
gcn5/EQ
Δgcn5, nse3/RE
gcn5/EQ, nse3/RE
E.
Ctrl (25°C)
37°C
5mM HU
0.01% MMS
WT
N4-FLAG
gcn5/EQ
gcn5/EQ, N4-FLAG

Figure S4

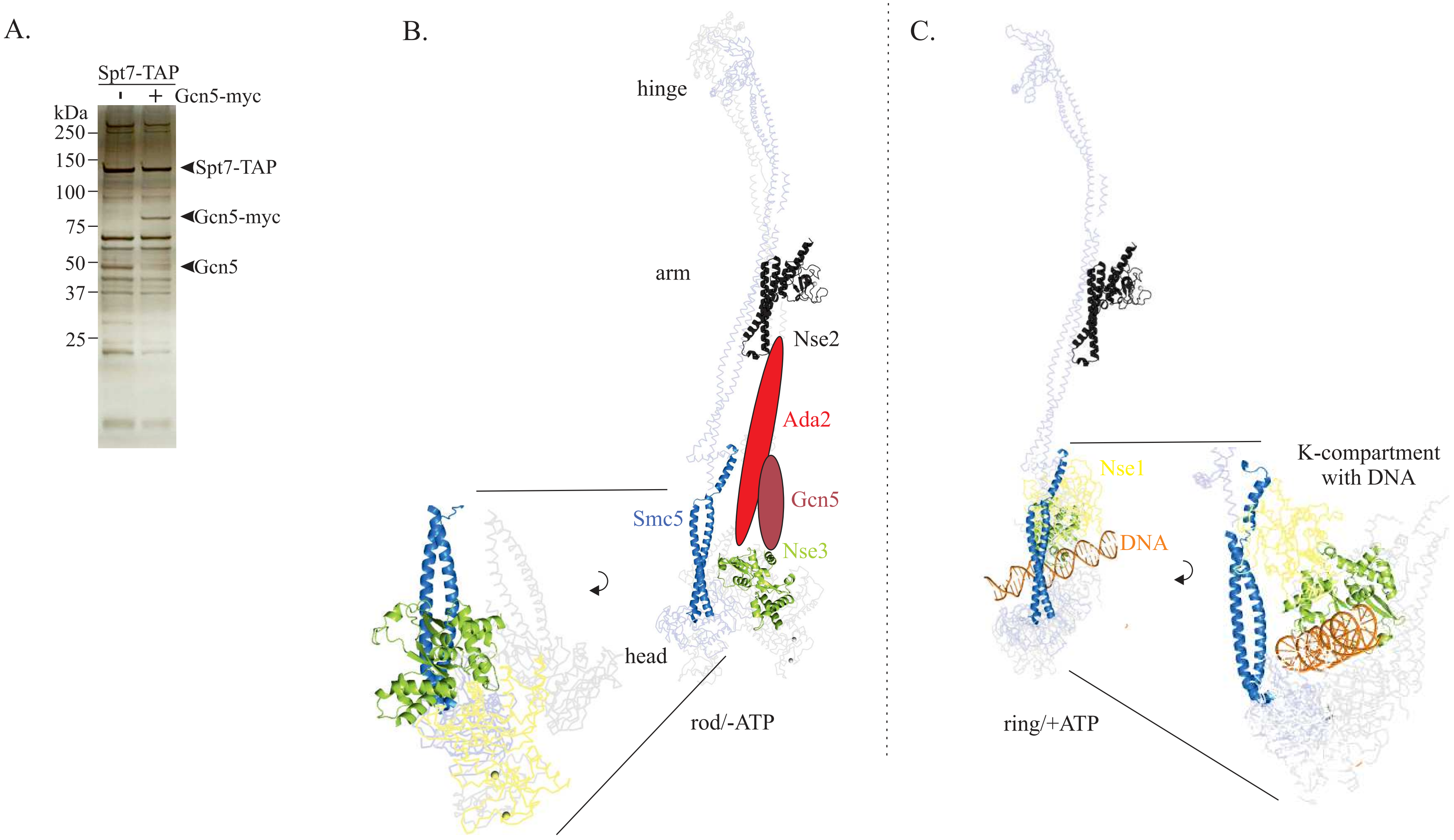
A.
Spt7-TAP
- + Gcn5-myc
kDa
250
150
100
75
50
37
25
◀Spt7-TAP
◀Gcn5-myc
◀Gcn5
B.
hinge
arm
Nse2
Ada2
Gcn5
Smc5
Nse3
head
rod/-ATP
C.
Nse1
K-compartment
with DNA
DNA
ring/+ATP

Table S1 - SGA scores

POMBASE ID	average score
ada2	-0,701
ade8	-0,727
alg14	-0,429
alp14	-0,494
**apn2**	-1,071
**ase1**	-0,339
atg22	-0,422
bdc1	-0,381
bdf1	-0,393
**brc1**	-0,666
**ccr4**	-0,553
cdr2	-0,646
cdt2	-0,441
cgs1	-1,095
dbl2	-0,582
**dbl8**	-0,360
dbs2	-0,388
**dcd1**	-0,500
dcn1	-0,330
ddb1	-0,462
eaf7	-0,337
ect1	-0,671
**elc1**	-0,303
**fft3**	-0,354
flx1	-0,792
gcn5	-0,545
**hrq1**	-0,760
ies6	-0,906
lsk1	-0,486
lsm1	-0,512
mcp6	-0,791
mpd2	-0,695
**mrc1**	-0,571
mtq1	-0,344
mub1	-0,436
myh1	-0,504
nem1	-0,766
nem2	-0,527
ngg1	-0,446
nrl1	-0,518
**nse5**	-0,756
**nse6**	-0,684
nth1	-0,695
nup132	-0,919
nup60	-0,546
png1	-0,487
**pnk1**	-0,387
pph3	-0,393
ppn1	-0,462
**rad2**	-0,418
**rad26**	-0,535
rad50	-0,526
rds1	-0,497
rei1	-0,391
**rhp14**	-0,726
**rhp41**	-0,950
rnh201	-0,323
rnh202	-0,393
rsm1	-0,852
set1	-0,300
SPAC1039.08	-0,656
SPAC15E1.02c	-0,516
SPAC513.04	-1,208
SPAC56F8.02	-0,515
SPAC8C9.04	-0,301
SPBC557.02c	-0,650
SPCC23B6.01c	-0,396
SPCC594.01	-0,762
srb11	-0,290
**srs2**	-0,842
tdh1	-0,485
**tdp1**	-0,370
tel1	-0,285
tsc2	-0,500
ubi4	-0,901
ubp8	-0,481
ubr1	-0,260
ulp2	-0,712
yox1	-0,381

bold: negative genetic interactions already described in previous studies

italics: genetic interactions verified by tetrad analysis (Suppl. Fig. S1B)

Table S2 - GO Biological process

p-value	GO-ID	Description	Genes in test set
3,27E-13	6259	DNA metabolic process	*SPBC3D6.10, SPAC17H9.19C, SPAC26A3.02, SPAC3G6.06C, SPCC24B10.08C, SPAC30D11.07, SPAC11E3.08C, SPAC4H3.05, SPCC553.01C, SPAC1556.01C, SPAC12B10.12C, SPCP31B10.05, SPBC582.06C, SPAC23C11.04C, SPCC23B6.03C, SPBC1347.08C, SPAC17H9.10C, SPBC649.03, SPCC306.04C, SPBC16A3.19, SPAC1952.05, SPBC651.10, SPAC4G9.02, SPAC3G9.08, SPBC582.05C*
1,69E-12	6974	response to DNA damage stimulus	*SPAC9E9.08, SPBC3D6.10, SPAC17H9.19C, SPAC17H9.10C, SPAC26A3.02, SPBC649.03, SPCC306.04C, SPAC3G6.06C, SPBC16A3.19, SPAC694.06C, SPBC651.10, SPAC30D11.07, SPAC11E3.08C, SPAC4H3.05, SPAC1556.01C, SPAC12B10.12C, SPAC3G9.08, SPCP31B10.05, SPAC23C11.04C, SPCC23B6.03C, SPBC582.05C*
9,06E-09	6950	response to stress	*SPAC343.12, SPAC9E9.08, SPBC3D6.10, SPAC17H9.19C, SPAC26A3.02, SPAC2G11.13, SPAC3G6.06C, SPCC24B10.08C, SPAC167.05, SPAC30D11.07, SPAC11E3.08C, SPAC8C9.03, SPAC4H3.05, SPAC1556.01C, SPAC12B10.12C, SPCP31B10.05, SPAC23C11.04C, SPCC23B6.03C, SPAC630.13C, SPCC23B6.01C, SPAC17H9.10C, SPBC649.03, SPCC306.04C, SPAC15E1.02C, SPBC16A3.19, SPAC694.06C, SPBC651.10, SPBC3B8.10C, SPAC3G9.08, SPBC582.05C, SPBC32F12.11*
1,77E-08	33554	cellular response to stress	*SPAC343.12, SPAC9E9.08, SPBC3D6.10, SPAC17H9.19C, SPAC26A3.02, SPAC2G11.13, SPAC3G6.06C, SPAC167.05, SPAC30D11.07, SPAC11E3.08C, SPAC8C9.03, SPAC4H3.05, SPAC1556.01C, SPAC12B10.12C, SPCP31B10.05, SPAC23C11.04C, SPCC23B6.03C, SPAC630.13C, SPCC23B6.01C, SPAC17H9.10C, SPBC649.03, SPCC306.04C, SPAC15E1.02C, SPBC16A3.19, SPAC694.06C, SPBC651.10, SPBC3B8.10C, SPAC3G9.08, SPBC582.05C, SPBC32F12.11*
4,47E-08	6281	DNA repair	*SPBC3D6.10, SPAC17H9.10C, SPAC26A3.02, SPBC649.03, SPAC3G6.06C, SPBC16A3.19, SPAC30D11.07, SPAC4H3.05, SPAC1556.01C, SPAC12B10.12C, SPAC3G9.08, SPCP31B10.05, SPAC23C11.04C, SPBC582.05C*
7,09E-08	51716	cellular response to stimulus	*SPAC343.12, SPAC9E9.08, SPBC3D6.10, SPAC17H9.19C, SPAC26A3.02, SPAC2G11.13, SPAC3G6.06C, SPAC167.05, SPAC30D11.07, SPAC11E3.08C, SPAC8C9.03, SPAC4H3.05, SPAC1556.01C, SPAC12B10.12C, SPCP31B10.05, SPAC23C11.04C, SPCC23B6.03C, SPAC630.13C, SPCC23B6.01C, SPAC17H9.10C, SPBC649.03, SPCC306.04C, SPAC15E1.02C, SPBC16A3.19, SPAC694.06C, SPBC651.10, SPBC3B8.10C, SPAC3G9.08, SPBC582.05C, SPBC32F12.11*
1,14E-07	50896	response to stimulus	*SPAC343.12, SPAC9E9.08, SPBC3D6.10, SPAC17H9.19C, SPAC26A3.02, SPAC2G11.13, SPAC3G6.06C, SPCC24B10.08C, SPAC167.05, SPAC30D11.07, SPAC11E3.08C, SPAC8C9.03, SPAC4H3.05, SPAC1556.01C, SPAC12B10.12C, SPCP31B10.05, SPAC23C11.04C, SPCC23B6.03C, SPAC630.13C, SPCC23B6.01C, SPAC17H9.10C, SPBC649.03, SPCC306.04C, SPAC15E1.02C, SPBC16A3.19, SPAC694.06C, SPBC651.10, SPBC3B8.10C, SPAC3G9.08, SPBC582.05C, SPBC32F12.11*

1,79E-07	75	cell cycle checkpoint	*SPCC895.07, SPAC1805.04, SPAC9E9.08, SPAC17H9.19C, SPAC2F3.15, SPAPB1A10.09, SPAC1556.01C, SPAC26A3.02, SPCC306.04C, SPCC23B6.03C, SPAC694.06C*
1,66E-06	43687	post-translational protein modification	*SPBC21D10.10, SPAC17H9.19C, SPBC337.08C, SPBC1861.07, SPAC17H9.10C, SPAC13A11.04C, SPBC31F10.10C, SPCC306.04C, SPBC16A3.19, SPAC1952.05, SPBC28F2.10C, SPCC24B10.08C, SPBC3B8.10C, SPAC29B12.05C, SPAC2F3.15, SPAC57A10.02, SPBC26H8.05C, SPBC19C7.02, SPBC839.03C, SPAC17A5.07C, SPCC23B6.03C*
2,04E-06	77	DNA damage checkpoint	*SPAC9E9.08, SPAC17H9.19C, SPAC1556.01C, SPAC26A3.02, SPCC306.04C, SPCC23B6.03C, SPAC694.06C*
2,85E-06	42770	DNA damage response, signal transduction	*SPAC9E9.08, SPAC17H9.19C, SPAC1556.01C, SPAC26A3.02, SPCC306.04C, SPCC23B6.03C, SPAC694.06C*
6,16E-06	90304	nucleic acid metabolic process	*SPCC74.02C, SPBC3D6.10, SPAC17H9.19C, SPAC26A3.02, SPBC29A10.10C, SPAC3G6.06C, SPCC24B10.08C, SPAC30D11.07, SPAC11E3.08C, SPAC4H3.05, SPCC553.01C, SPAC1556.01C, SPAC12B10.12C, SPCP31B10.05, SPBC582.06C, SPAC23C11.04C, SPCC23B6.03C, SPBC12D12.06, SPBC1861.07, SPBC1347.08C, SPAC17H9.10C, SPAC13A11.04C, SPBC649.03, SPCC306.04C, SPBC16A3.19, SPAC1952.05, SPBC28F2.10C, SPBC651.10, SPBC3D6.08C, SPAC4G9.02, SPCC31H12.08C, SPAC3G9.08, SPBC582.05C*
7,32E-06	51276	chromosome organization	*SPBC21D10.10, SPAC13A11.04C, SPCC1450.02, SPBC649.03, SPCC306.04C, SPAC222.04C, SPAC3G6.06C, SPBC16A3.19, SPAC1952.05, SPBC28F2.10C, SPCC24B10.08C, SPAC25A8.01C, SPCC553.01C, SPAC1556.01C, SPAC17A5.07C, SPBC582.06C, SPCC23B6.03C, SPBC582.05C*
9,34E-06	51052	regulation of DNA metabolic process	*SPBC651.10, SPAC9E9.08, SPAC17H9.19C, SPAC11E3.08C, SPAC17H9.10C, SPAC3G9.08, SPBC582.05C, SPAC694.06C*
1,21E-05	6139	nucleobase, nucleoside, nucleotide and nucleic acid metabolic process	*SPCC74.02C, SPBC3D6.10, SPAC17H9.19C, SPAC26A3.02, SPBC29A10.10C, SPAC3G6.06C, SPCC24B10.08C, SPAC30D11.07, SPAC11E3.08C, SPAC4H3.05, SPCC553.01C, SPAC1556.01C, SPAC12B10.12C, SPCP31B10.05, SPBC582.06C, SPAC23C11.04C, SPCC23B6.03C, SPBC12D12.06, SPBC14F5.09C, SPBC1861.07, SPBC1347.08C, SPAC17H9.10C, SPAC13A11.04C, SPBC649.03, SPCC306.04C, SPBC16A3.19, SPAC1952.05, SPBC28F2.10C, SPBC2G2.13C, SPBC651.10, SPBC3D6.08C, SPAC4G9.02, SPCC31H12.08C, SPAC3G9.08, SPBC582.05C*
1,98E-05	31570	DNA integrity checkpoint	*SPAC9E9.08, SPAC17H9.19C, SPAC1556.01C, SPAC26A3.02, SPCC306.04C, SPCC23B6.03C, SPAC694.06C*
2,50E-05	16573	histone acetylation	*SPCC24B10.08C, SPBC21D10.10, SPAC13A11.04C, SPBC16A3.19, SPAC1952.05, SPBC28F2.10C*
3,35E-05	6464	protein modification process	*SPBC21D10.10, SPAC17H9.19C, SPBC337.08C, SPBC1861.07, SPAC17H9.10C, SPAC13A11.04C, SPBC31F10.10C, SPCC306.04C, SPBC16A3.19, SPAC1952.05, SPBC28F2.10C, SPCC24B10.08C, SPBC3B8.10C, SPAC29B12.05C, SPAC2F3.15, SPAC57A10.02, SPBC26H8.05C, SPBC19C7.02, SPBC839.03C, SPAC17A5.07C, SPCC23B6.03C, SPAC5D6.06C*

6,27E-05	51327	M phase of meiotic cell cycle	*SPCC24B10.08C, SPAC167.05, SPBC902.03, SPBC337.08C, SPAC8C9.04, SPAPB1A10.09, SPCC553.01C, SPAC1556.01C, SPBC31F10.10C, SPBC582.06C, SPAC1952.05*
6,27E-05	7126	meiosis	*SPCC24B10.08C, SPAC167.05, SPBC902.03, SPBC337.08C, SPAC8C9.04, SPAPB1A10.09, SPCC553.01C, SPAC1556.01C, SPBC31F10.10C, SPBC582.06C, SPAC1952.05*
6,62E-05	51726	regulation of cell cycle	*SPAC1805.04, SPAC9E9.08, SPAC17H9.19C, SPAC17H9.10C, SPAC26A3.02, SPCC306.04C, SPAC694.06C, SPCC895.07, SPAC2F3.15, SPAC8C9.03, SPAC57A10.02, SPAPB1A10.09, SPAC1556.01C, SPCC23B6.03C*
7,36E-05	34641	cellular nitrogen compound metabolic process	*SPCC74.02C, SPBC3D6.10, SPAC17H9.19C, SPAC26A3.02, SPBC29A10.10C, SPAC3G6.06C, SPCC24B10.08C, SPAC30D11.07, SPAC11E3.08C, SPAC4H3.05, SPCC553.01C, SPAC1556.01C, SPAC12B10.12C, SPCP31B10.05, SPBC582.06C, SPAC23C11.04C, SPCC23B6.03C, SPBC12D12.06, SPBC14F5.09C, SPBC1861.07, SPBC1347.08C, SPAC17H9.10C, SPAC13A11.04C, SPBC649.03, SPCC306.04C, SPBC16A3.19, SPAC1952.05, SPBC28F2.10C, SPBC2G2.13C, SPBC651.10, SPAC15E1.05C, SPBC3D6.08C, SPAC4G9.02, SPCC31H12.08C, SPAC1039.08, SPAC3G9.08, SPBC582.05C*
7,68E-05	51321	meiotic cell cycle	*SPCC24B10.08C, SPAC167.05, SPBC902.03, SPBC337.08C, SPAC8C9.04, SPAPB1A10.09, SPCC553.01C, SPAC1556.01C, SPBC31F10.10C, SPBC582.06C, SPAC1952.05*
8,54E-05	6807	nitrogen compound metabolic process	*SPCC74.02C, SPBC3D6.10, SPAC17H9.19C, SPAC26A3.02, SPBC29A10.10C, SPAC3G6.06C, SPCC24B10.08C, SPAC30D11.07, SPAC11E3.08C, SPAC4H3.05, SPCC553.01C, SPAC1556.01C, SPAC12B10.12C, SPCP31B10.05, SPBC582.06C, SPAC23C11.04C, SPCC23B6.03C, SPBC12D12.06, SPBC14F5.09C, SPBC1861.07, SPBC1347.08C, SPAC17H9.10C, SPAC13A11.04C, SPBC649.03, SPCC306.04C, SPBC16A3.19, SPAC1952.05, SPBC28F2.10C, SPBC2G2.13C, SPBC651.10, SPAC15E1.05C, SPBC3D6.08C, SPAC4G9.02, SPCC31H12.08C, SPAC1039.08, SPAC3G9.08, SPBC582.05C*
1,15E-04	6260	DNA replication	*SPBC651.10, SPAC4G9.02, SPAC17H9.19C, SPAC11E3.08C, SPBC1347.08C, SPAC17H9.10C, SPAC4H3.05, SPAC1556.01C, SPAC3G6.06C*
1,20E-04	6338	chromatin remodeling	*SPCC24B10.08C, SPBC21D10.10, SPAC25A8.01C, SPAC13A11.04C, SPCC1450.02, SPCC306.04C, SPAC222.04C, SPBC16A3.19, SPAC1952.05, SPBC28F2.10C*
1,41E-04	16569	covalent chromatin modification	*SPCC24B10.08C, SPBC21D10.10, SPAC13A11.04C, SPCC306.04C, SPCC23B6.03C, SPBC16A3.19, SPAC1952.05, SPBC28F2.10C*
1,41E-04	16570	histone modification	*SPCC24B10.08C, SPBC21D10.10, SPAC13A11.04C, SPCC306.04C, SPCC23B6.03C, SPBC16A3.19, SPAC1952.05, SPBC28F2.10C*
1,42E-04	6473	protein amino acid acetylation	*SPCC24B10.08C, SPBC21D10.10, SPAC13A11.04C, SPBC16A3.19, SPAC1952.05, SPBC28F2.10C*
1,50E-04	16568	chromatin modification	*SPCC24B10.08C, SPBC21D10.10, SPAC25A8.01C, SPAC13A11.04C, SPCC1450.02, SPCC306.04C, SPAC222.04C, SPCC23B6.03C, SPBC16A3.19, SPAC1952.05, SPBC28F2.10C*

1,95E-04	50789	regulation of biological process	*SPBC21B10.13C, SPAC9E9.08, SPAC17H9.19C, SPAC26A3.02, SPCC24B10.08C, SPAC11E3.08C, SPAC25A8.01C, SPAC8C9.03, SPAC57A10.02, SPAC1556.01C, SPBC19C7.02, SPCC23B6.03C, SPAC630.13C, SPAC1805.04, SPBC1861.07, SPAC17H9.10C, SPCC306.04C, SPBC16A3.19, SPAC694.06C, SPAC1952.05, SPCC895.07, SPBC651.10, SPBC3B8.10C, SPBC902.03, SPAC2F3.15, SPAPB1A10.09, SPBC26H8.05C, SPAC3G9.08, SPBC839.03C, SPBC582.05C, SPBC32F12.11*
2,17E-04	43412	macromolecule modification	*SPBC21D10.10, SPAC17H9.19C, SPBC337.08C, SPBC1861.07, SPAC17H9.10C, SPAC13A11.04C, SPBC31F10.10C, SPCC306.04C, SPBC16A3.19, SPAC1952.05, SPBC28F2.10C, SPCC24B10.08C, SPBC3B8.10C, SPAC29B12.05C, SPAC2F3.15, SPAC57A10.02, SPBC26H8.05C, SPBC19C7.02, SPBC839.03C, SPAC17A5.07C, SPCC23B6.03C, SPAC5D6.06C*
2,61E-04	6284	base-excision repair	*SPAC30D11.07, SPBC3D6.10, SPAC26A3.02*
3,08E-04	43543	protein amino acid acylation	*SPCC24B10.08C, SPBC21D10.10, SPAC13A11.04C, SPBC16A3.19, SPAC1952.05, SPBC28F2.10C*
3,65E-04	6325	chromatin organization	*SPCC24B10.08C, SPBC21D10.10, SPAC25A8.01C, SPAC13A11.04C, SPCC1450.02, SPCC306.04C, SPAC222.04C, SPCC23B6.03C, SPBC16A3.19, SPAC1952.05, SPBC28F2.10C*
3,69E-04	80135	regulation of cellular response to stress	*SPAC17H9.10C, SPAC3G9.08, SPBC32F12.11*
3,69E-04	71478	cellular response to radiation	*SPAC17H9.10C, SPAC4H3.05, SPAC26A3.02*
3,69E-04	71482	cellular response to light stimulus	*SPAC17H9.10C, SPAC4H3.05, SPAC26A3.02*
3,69E-04	34644	cellular response to UV	*SPAC17H9.10C, SPAC4H3.05, SPAC26A3.02*
4,62E-04	23046	signaling process	*SPAC1805.04, SPAC9E9.08, SPAC17H9.19C, SPAC26A3.02, SPCC306.04C, SPAC694.06C, SPCC895.07, SPAC8C9.03, SPAC57A10.02, SPBC26H8.05C, SPAC1556.01C, SPCC23B6.03C, SPBC32F12.11, SPAC630.13C*
4,62E-04	23060	signal transmission	*SPAC1805.04, SPAC9E9.08, SPAC17H9.19C, SPAC26A3.02, SPCC306.04C, SPAC694.06C, SPCC895.07, SPAC8C9.03, SPAC57A10.02, SPBC26H8.05C, SPAC1556.01C, SPCC23B6.03C, SPBC32F12.11, SPAC630.13C*
4,62E-04	7165	signal transduction	*SPAC1805.04, SPAC9E9.08, SPAC17H9.19C, SPAC26A3.02, SPCC306.04C, SPAC694.06C, SPCC895.07, SPAC8C9.03, SPAC57A10.02, SPBC26H8.05C, SPAC1556.01C, SPCC23B6.03C, SPBC32F12.11,*
5,02E-04	80134	regulation of response to stress	*SPAC17H9.10C, SPAC3G9.08, SPBC32F12.11*
5,85E-04	8156	negative regulation of DNA replication	*SPBC651.10, SPAC9E9.08, SPAC17H9.19C, SPAC11E3.08C, SPAC694.06C*
6,16E-04	70647	protein modification by small protein conjugation or removal	*SPAC17H9.19C, SPBC337.08C, SPBC1861.07, SPAC17H9.10C, SPAC13A11.04C, SPBC19C7.02, SPBC31F10.10C, SPBC839.03C, SPAC17A5.07C*
6,62E-04	43137	DNA replication, removal of RNA primer	*SPAC4G9.02, SPBC1347.08C, SPAC3G6.06C*
7,50E-04	6289	nucleotide-excision repair	*SPAC17H9.10C, SPAC12B10.12C, SPBC649.03, SPAC23C11.04C*

7,59E-04	44265	cellular macromolecule catabolic process	*SPAC30D11.07, SPBC3D6.08C, SPAC4G9.02, SPAC17H9.19C, SPBC1861.07, SPBC1347.08C, SPAC17H9.10C, SPAC13A11.04C, SPCC31H12.08C, SPBC19C7.02, SPAC23C11.04C, SPAC3G6.06C*
7,84E-04	279	M phase	*SPCC24B10.08C, SPAC167.05, SPCC895.07, SPBC902.03, SPBC337.08C, SPAC8C9.04, SPAPB1A10.09, SPCC553.01C, SPAC1556.01C, SPBC31F10.10C, SPBC582.06C, SPAC1952.05*
7,95E-04	65007	biological regulation	*SPBC21B10.13C, SPAC9E9.08, SPAC17H9.19C, SPAC26A3.02, SPAC3G6.06C, SPCC24B10.08C, SPAC11E3.08C, SPAC25A8.01C, SPAC8C9.03, SPAC57A10.02, SPCC553.01C, SPAC1556.01C, SPBC19C7.02, SPCC23B6.03C, SPAC630.13C, SPAC1805.04, SPBC1861.07, SPAC17H9.10C, SPCC306.04C, SPBC16A3.19, SPAC694.06C, SPAC1952.05, SPCC895.07, SPBC651.10, SPBC3B8.10C, SPBC902.03, SPAC2F3.15, SPAPB1A10.09, SPBC26H8.05C, SPAC3G9.08, SPBC839.03C, SPBC582.05C, SPBC32F12.11*
7,99E-04	23052	signaling	*SPAC1805.04, SPAC9E9.08, SPAC17H9.19C, SPAC26A3.02, SPCC306.04C, SPAC694.06C, SPCC895.07, SPAC8C9.03, SPAC57A10.02, SPBC26H8.05C, SPAC1556.01C, SPCC23B6.03C, SPBC32F12.11, SPAC630.13C*
8,51E-04	9411	response to UV	*SPAC17H9.10C, SPAC4H3.05, SPAC26A3.02*
8,51E-04	9416	response to light stimulus	*SPAC17H9.10C, SPAC4H3.05, SPAC26A3.02*
8,51E-04	48583	regulation of response to stimulus	*SPAC17H9.10C, SPAC3G9.08, SPBC32F12.11*
8,83E-04	22403	cell cycle phase	*SPAC9E9.08, SPBC337.08C, SPBC31F10.10C, SPAC1952.05, SPCC24B10.08C, SPAC167.05, SPCC895.07, SPBC902.03, SPAC8C9.04, SPAPB1A10.09, SPCC553.01C, SPAC1556.01C, SPBC582.06C*
9,44E-04	51053	negative regulation of DNA metabolic process	*SPBC651.10, SPAC9E9.08, SPAC17H9.19C, SPAC11E3.08C, SPAC694.06C*
1,07E-03	9314	response to radiation	*SPAC17H9.10C, SPAC4H3.05, SPAC26A3.02*
1,07E-03	71214	cellular response to abiotic stimulus	*SPAC17H9.10C, SPAC4H3.05, SPAC26A3.02*
1,07E-03	33567	DNA replication, Okazaki fragment	*SPAC4G9.02, SPBC1347.08C, SPAC3G6.06C*
1,19E-03	6261	DNA-dependent DNA replication	*SPBC651.10, SPAC4G9.02, SPAC17H9.19C, SPAC11E3.08C, SPBC1347.08C, SPAC4H3.05, SPAC3G6.06C*
1,32E-03	6273	lagging strand elongation	*SPAC4G9.02, SPBC1347.08C, SPAC3G6.06C*
1,32E-03	31573	intra-S DNA damage checkpoint	*SPAC9E9.08, SPAC1556.01C, SPAC694.06C*
1,33E-03	6275	regulation of DNA replication	*SPBC651.10, SPAC9E9.08, SPAC17H9.19C, SPAC11E3.08C, SPAC694.06C*
1,33E-03	32200	telomere organization	*SPCC553.01C, SPAC1556.01C, SPCC306.04C, SPCC23B6.03C, SPAC3G6.06C*
1,33E-03	723	telomere maintenance	*SPCC553.01C, SPAC1556.01C, SPCC306.04C, SPCC23B6.03C, SPAC3G6.06C*
1,33E-03	60249	anatomical structure homeostasis	*SPCC553.01C, SPAC1556.01C, SPCC306.04C, SPCC23B6.03C, SPAC3G6.06C*
1,49E-03	9057	macromolecule catabolic process	*SPAC30D11.07, SPBC3D6.08C, SPAC4G9.02, SPAC17H9.19C, SPBC1861.07, SPBC1347.08C, SPAC17H9.10C, SPAC13A11.04C, SPCC31H12.08C, SPBC19C7.02, SPAC23C11.04C, SPAC3G6.06C*

1,51E-03	22402	cell cycle process	*SPAC9E9.08, SPBC337.08C, SPBC31F10.10C, SPAC1952.05, SPCC24B10.08C, SPAC167.05, SPCC895.07,*
1,74E-03	50794	regulation of cellular process	*SPBC21B10.13C, SPAC9E9.08, SPAC17H9.19C, SPAC26A3.02, SPCC24B10.08C, SPAC11E3.08C, SPAC25A8.01C, SPAC8C9.03, SPAC57A10.02, SPAC1556.01C, SPCC23B6.03C, SPAC630.13C, SPAC1805.04, SPBC1861.07, SPAC17H9.10C, SPCC306.04C, SPBC16A3.19, SPAC694.06C, SPAC1952.05, SPCC895.07, SPBC651.10, SPAC2F3.15, SPAPB1A10.09, SPBC26H8.05C, SPAC3G9.08, SPBC839.03C, SPBC582.05C, SPBC32F12.11*
2,19E-03	6282	regulation of DNA repair	*SPAC17H9.10C, SPAC3G9.08*
2,19E-03	31565	cytokinesis checkpoint	*SPAC2F3.15, SPAPB1A10.09*
2,49E-03	23034	intracellular signaling pathway	*SPAC9E9.08, SPAC17H9.19C, SPAC1556.01C, SPAC26A3.02, SPCC306.04C, SPCC23B6.03C, SPBC32F12.11, SPAC630.13C, SPAC694.06C*
2,76E-03	80090	regulation of primary metabolic process	*SPBC21B10.13C, SPAC9E9.08, SPAC17H9.19C, SPBC1861.07, SPAC17H9.10C, SPBC16A3.19, SPAC694.06C, SPAC1952.05, SPCC24B10.08C, SPBC651.10, SPBC3B8.10C, SPBC902.03, SPAC11E3.08C, SPAC2F3.15, SPAC8C9.03, SPAC3G9.08, SPBC839.03C, SPBC582.05C, SPAC630.13C*
2,98E-03	6996	organelle organization	*SPBC21D10.10, SPAC1805.04, SPAC13A11.04C, SPCC1450.02, SPBC649.03, SPCC306.04C, SPAC222.04C, SPAC3G6.06C, SPBC16A3.19, SPAC1952.05, SPBC28F2.10C, SPCC24B10.08C, SPCC895.07, SPBC3B8.10C, SPBC902.03, SPAC29B12.05C, SPAC25A8.01C, SPAPB1A10.09, SPCC553.01C, SPAC1556.01C, SPAC17A5.07C, SPBC582.06C, SPCC23B6.03C, SPBC582.05C*
3,63E-03	44093	positive regulation of molecular function	*SPAC17H9.19C, SPBC839.03C, SPAC630.13C*
3,63E-03	43085	positive regulation of catalytic activity	*SPAC17H9.19C, SPBC839.03C, SPAC630.13C*
3,72E-03	23033	signaling pathway	*SPAC9E9.08, SPAC17H9.19C, SPAC1556.01C, SPAC26A3.02, SPCC306.04C, SPCC23B6.03C, SPBC32F12.11, SPAC630.13C, SPAC694.06C*
4,03E-03	32446	protein modification by small protein conjugation	*SPAC17H9.19C, SPBC337.08C, SPBC1861.07, SPAC17H9.10C, SPBC19C7.02, SPBC31F10.10C, SPBC839.03C*
4,50E-03	51443	positive regulation of ubiquitin-protein ligase activity	*SPAC17H9.19C, SPBC839.03C*
4,50E-03	31398	positive regulation of protein ubiquitination	*SPAC17H9.19C, SPBC839.03C*
4,61E-03	7049	cell cycle	*SPAC9E9.08, SPBC337.08C, SPBC31F10.10C, SPAC1952.05, SPCC24B10.08C, SPAC167.05, SPCC895.07, SPBC902.03, SPAC2F3.15, SPAC8C9.04, SPAPB1A10.09, SPCC553.01C, SPAC1556.01C, SPBC582.06C*
5,33E-03	19222	regulation of metabolic process	*SPBC21B10.13C, SPAC9E9.08, SPAC17H9.19C, SPBC1861.07, SPAC17H9.10C, SPBC16A3.19, SPAC694.06C, SPAC1952.05, SPCC24B10.08C, SPBC651.10, SPBC3B8.10C, SPBC902.03, SPAC11E3.08C,*

5,67E-03	7131	reciprocal meiotic recombination	*SPCC24B10.08C, SPAC1556.01C, SPBC582.06C, SPAC1952.05*
5,95E-03	51351	positive regulation of ligase activity	*SPAC17H9.19C, SPBC839.03C*
5,95E-03	46579	positive regulation of Ras protein signal transduction	*SPAC2F3.15, SPAC25A8.01C*
5,95E-03	46890	regulation of lipid biosynthetic process	*SPBC3B8.10C, SPBC902.03*
5,95E-03	51057	positive regulation of small GTPase mediated signal transduction	*SPAC2F3.15, SPAC25A8.01C*
6,01E-03	70646	protein modification by small protein removal	*SPBC337.08C, SPAC13A11.04C, SPAC17A5.07C*
6,26E-03	19219	regulation of nucleobase, nucleoside, nucleotide and nucleic acid metabolic process	*SPBC21B10.13C, SPAC9E9.08, SPAC17H9.19C, SPBC1861.07, SPAC17H9.10C, SPBC16A3.19, SPAC694.06C, SPAC1952.05, SPCC24B10.08C, SPBC651.10, SPAC11E3.08C, SPAC2F3.15, SPAC3G9.08, SPBC582.05C, SPAC630.13C*
6,53E-03	6401	RNA catabolic process	*SPBC3D6.08C, SPAC4G9.02, SPBC1347.08C, SPCC31H12.08C, SPAC3G6.06C*
6,87E-03	51171	regulation of nitrogen compound metabolic process	*SPBC21B10.13C, SPAC9E9.08, SPAC17H9.19C, SPBC1861.07, SPAC17H9.10C, SPBC16A3.19, SPAC694.06C, SPAC1952.05, SPCC24B10.08C, SPBC651.10, SPAC11E3.08C, SPAC2F3.15, SPAC3G9.08, SPBC582.05C, SPAC630.13C*

Table S3 - GO Cellular Component

p-value	GO-ID	Description	Genes in test set
9,86E-07	123	histone acetyltransferase complex	*ada2, ada3, bdc1, eaf7, gcn5, png1, ubp8*
3,38E-06	5634	nucleus	*ada2, ada3, ade8, alp14, apn2, bdc1, bdf1, brc1, ccr4, cdr2, cdt2, cgs1, dbl2, dbl8, dcd1, dcn1, ddb1, eaf7, ect1, fft3, gcn5, hrq1, ies6, lsk1, lsm1, mcp6, mrc1, mub1, myh1, nrl1, nse5, nse6, nth1, nup132, nup60, png1, pnk1, ppe2, ppn1, rad2, rad26, rad50, rhp14, rhp41, rnh201, rsm1, set1, SPAC1039.08, SPBC1347.08C, SPBC1861.07, SPBC557.02C, SPCC23B6.01C, srb11, srs2, tdp1, tsc2, ubp8, ubr1, ulp2, yox1*
6,71E-05	43234	protein complex	*ada2, ada3, alp14, ase1, bdc1, bdf1, ccr4, cdr2, cgs1, ddb1, eaf7, fft3, gcn5, ies6, lsk1, lsm1, mrc1, nem1, nse5, nse6, nup132, nup60, png1, rad50, rhp14, rhp41, rnh201, set1, SPBC1347.08C, SPBC1861.07, SPBC902.03, srb11, tsc2, ubp8, yox1*
1,13E-04	44451	nucleoplasm part	*ada2, ada3, bdc1, ccr4, eaf7, gcn5, lsk1, png1, set1, SPBC1861.07, srb11, ubp8, yox1*
1,58E-04	70461	SAGA-type complex	*ada2, ada3, gcn5, ubp8*
1,58E-04	124	SAGA complex	*ada2, ada3, gcn5, ubp8*
1,84E-04	5654	nucleoplasm	*ada2, ada3, bdc1, ccr4, eaf7, gcn5, lsk1, png1, set1, SPBC1861.07, srb11, ubp8, yox1*
3,78E-04	44427	chromosomal part	*ada2, ada3, alp14, bdc1, bdf1, eaf7, fft3, gcn5, ies6, mrc1, nse5, nse6, rad2, rad50, set1, tel1*
5,61E-04	790	nuclear chromatin	*ada2, ada3, bdc1, bdf1, eaf7, fft3, gcn5, ies6, mrc1, rad50, set1*
6,07E-04	5694	chromosome	*ada2, ada3, alp14, bdc1, bdf1, eaf7, fft3, gcn5, ies6, mrc1, nse5, nse6, rad2, rad50, set1, tel1*
6,70E-04	32299	ribonuclease H2 complex	*rnh201, SPBC1347.08C*
8,53E-04	35267	NuA4 histone acetyltransferase complex	*bdc1, eaf7, png1*
1,07E-03	43189	H4/H2A histone acetyltransferase complex	*bdc1, eaf7, png1*
1,32E-03	44454	nuclear chromosome part	*ada2, ada3, alp14, bdc1, bdf1, eaf7, fft3, gcn5, ies6, mrc1, rad2, rad50, set1*
1,76E-03	785	chromatin	*ada2, ada3, bdc1, bdf1, eaf7, fft3, gcn5, ies6, mrc1, rad50, set1*
2,12E-03	228	nuclear chromosome	*ada2, ada3, alp14, bdc1, bdf1, eaf7, fft3, gcn5, ies6, mrc1, rad2, rad50, set1*
3,15E-03	109	nucleotide-excision repair complex	*ddb1, rhp14, rhp41*
5,25E-03	5819	spindle	*alp14, ase1, cdr2, dbl2, mcp6, mub1, nse5, rhp41, SPAC56F8.02, srs2*
5,96E-03	30915	Smc5/6 complex	*nse5, nse6*

Table S4 - Cloning primers

pGADT7-spAda2	LJ121	fw	5’ GGAGGCCAGTGAATTCATGCCTCCTCAAAAGTATCATTGC 3’
	LJ122	rev	5’ CACCCGGGTGGAATTCTTAGGTTGGAGCGCCAATC 3’
pGBKT7-spAda2	LJ119	fw	5’ CATGGAGGCCGAATTCATGCCTCCTCAAAAGTATCATTGC 3’
	LJ120	rev	5’ GGATCCCCGGGAATTCTTAGGTTGGAGCGCCAATC 3’
pGBKT7-spGcn5	LJ101	fw	5’ GAATTCCCGGGGATCCCATCGAATTCGTTAAATGATCAAAC 3’
	LJ102	rev	5’ GCAGGTCGACGGATCCTTAATCGGCTAAGTGTGAATA 3’
pGBKT7-hTADA2B	LJ209	fw	5’ CATGGAGGCCGAATTCATGGCTGCCCACGTTG 3’
	LJ210	rev	5’ GGATCCCCGGGAATTCTCAAGACGCGTCCCTGG 3’
pGADT7-spNse3 (aa 1-90)	oPK171	fw	5’ GAACGGAATGAAACAGACGCAATAAATTTTTAACTATTGGTACGAAA 3’
	oPK172	rev	5’ TTTCGTACCAATAGTTAAAAATTTATTGCGTCTGTTTCATTCCGTTC 3’
pGADT7-spNse3 (aa 1-190)	oPK173	fw	5’ GTGTTGAGGTCTACCCTTCCTTAGGAACTTCAAAAGGATTC 3’
	oPK174	rev	5’ GAATCCTTTTGAAGTTCCTAAGGAAGGGTAGACCTCAACAC 3’
pGADT7-hNSE4a	oLV111	fw	5’ CAGATTACGCTCATATGTCTGGGGACAGCAGC 3’
	oLV112	rev	5’ TCATCTGCAGCTCGAGTCAAGCACTTGGCTTCTGC 3’
pGBKT7-spSMC5 (aa170-225+837-910)	JP125	fw	5’ GGGACCATGGCACATGAGAAGCTTATTGATTTA 3’
	JP126	rev	5’ GGAGCGGCCGCTAGTGATGGTGATGGTGATGAGAAAATCTATCGCTAATACATTG 3’
pGBKT7-spSMC5 (aa600-835)	JP295	fw	5’ GACGACGACAAGATGTACGGTGATAGAGAAAT 3’
	JP259	rev	5’ GAGGAGAAGCCCGGTTCACACTTCCGAACTAGTAGCAAA 3’
pGBKT7-spSMC6 (aa1-1140)	oLV465	fw	5’ AGGAGGACCTGCATATGATGACTACAGAGCTTACTAATGTATCT 3’
	oLV466	rev	5’ GCCGCTGCAGGTCGACTCATGGCGCCGTAGA 3’
pAW8-spNse3, *Sph* I to *Xho* I mutation	LJ48	fw	5’ CATACATTATACGAAGTTATCTCGAGGTGAATACGGTAGATAC 3’
	LJ49	rev	5’ GTATCTACCGTATTCACCTCGAGATAACTTCGTATAATGTATG 3’
pAW8-cloNAT-spNse3, cloNAT casette insertion	LJ42	fw	5’ ACGAAGTTATCTCGAGTGCTTTCTCAGGTATAGTATGAGGTC 3’
	LJ43	rev	5’ CCGTATTCACCTCGATGGAATTCGAGCTCGTTTAAACTGGATG 3’
pAW8-cloNAT-spNse3, 5’-end extension	LJ44	fw	5’ ACGAAGTTATCTCGAGACTAGTTAATTGACAGCTCAGCAATACAAC 3’
	LJ45	rev	5’ CCTGAGAAAGCACTCGAGTAACATTTATTACTATATTTCAGAGCAAACCTCC 3’

Table S5 - Yeast strains

Code	Genotype	Source
501	*h-, ura4-D18, ade6-704, leu1-32*	
503	*h+, ura4-D18, ade6-704, leu1-32*	
DHP260	*h+, gcn5-Myc13::natMX6, ura-D18, ade6-M216*	Helmlinger et al., 2008
DHP363	*h+, gcn5-1(E191Q)-MYC13::natMX6, ura4-D18, ade6-M216*	Helmlinger et al., 2008
DHP409	*h-, Δsus1::URA4, ura-D18, ade6-M210*	Helmlinger et al., 2011
DH783	*h-, spt7-HA3-TAP2::kanMX6*	Laboucarié et al., 2017
DH815	*h-, spt7-HA3-TAP2::kanMX6, gcn5-MYC13::natMX6*	Laboucarié et al., 2017
NBY772	*h-, nse4:GFP::HphMx4*	Pebernard et al., 2008
MA1-6	*h-, nse3-R254E::Lox, ura4-D18, ade6-704, leu1-32*	Zabrady et al., 2016
MA23	*h+, nse3-R254E::Lox, ura4-D18, ade6-704, leu1-32*	Zabrady et al., 2016
MMP21	*h-, nse4-FLAG-His::KanR leu1-32 ura4-D18*	Morikawa et al., 2004
JMM956	*h-, smc6-74, ade6-704, leu1-32, ura4-D18*	Verkade et al., 1999
JMM1920	*h-, smc6-X, ade6-704, leu1-32, ura4-D18*	Lehmann et al., 1995
JMM2050	*h+, nse2-SA::Ura4, leu1-32, ura4-d18,ade6-704*	Andrews et al., 2005
PEM2	*h-, mat1_m-cyhS, smt0, rpl42::cyhR (sP56Q), ade6-M210, leu1-32, ura4-D18*	Roguev et al., 2007
Δada2	*h+, Δada2::G418, ade6-216, ura4-D18, leu1-32*	BIONEER
Δada3	*h+, Δada3::G418, ade6-216, ura4-D18, leu1-32*	BIONEER
Δbdc1	*h+, Δbdc1::G418, ade6-210, ura4-D18, leu1-32*	BIONEER
Δbdf1	*h+, Δbdf1::G418, ade6-216, ura4-D18, leu1-32*	BIONEER
Δcdt2	*h+, Δcdt2::G418, ade6-216, ura4-D18, leu1-32*	BIONEER
Δdbl2	*h+, Δdbl2::G418, ade6-216, ura4-D18, leu1-32*	BIONEER
Δdcn1	*h+, Δdcn1::G418, ade6-216, ura4-D18, leu1-32*	BIONEER
Δddb1	*h+, Δddb1::G418, ade6-216, ura4-D18, leu1-32*	BIONEER
Δeaf7	*h+, Δeaf7::G418, ade6-216, ura4-D18, leu1-32*	BIONEER
Δgcn5	*h+, Δ gcn5::G418, ade6-216, ura4-D18, leu1-32*	BIONEER
Δnth1	*h+, Δnth1::G418, ade6-210, ura4-D18, leu1-32*	BIONEER
Δnup132	*h+, Δnup132::G418, ade6-216, ura4-D18, leu1-32*	BIONEER
Δnup60	*h+, Δnup60::G418, ade6-216, ura4-D18, leu1-32*	BIONEER

Δpng1	*h+, Δpng1::G418, ade6-216, ura4-D18, leu1-32*	BIONEER
Δrad50	*h+, Δrad50::G418, ade6-216, ura4-D18, leu1-32*	BIONEER
Δrnh201	*h+, Δrnh201::G418, ade6-216, ura4-D18, leu1-32*	BIONEER
Δsgf11	*h+, Δsgf11::G418, ade6-216, ura4-D18, leu1-32*	BIONEER
Δsgf29	*h+, Δsgf29::G418, ade6-210, ura4-D18, leu1-32*	BIONEER
Δsgf73	*h+, Δsgf73::G418, ade6-216, ura4-D18, leu1-32*	BIONEER
ΔSPBC1347.08c	*h+, ΔSPBC1347.08c::G418, ade6-210, ura4-D18, leu1-32*	BIONEER
Δtra1	*h+, Δtra1::G418, ade6-216, ura4-D18, leu1-32*	BIONEER
Δubp8	*h+, Δubp8::G418, ade6-216, ura4-D18, leu1-32*	BIONEER
Δubr1	*h+, Δubr1::G418, ade6-216, ura4-D18, leu1-32*	BIONEER
YLJ222	*h-, NAT-nse3, mat1_m-cyhS, smt0, rpl42::cyhR (sP56Q), ade6-M210, leu1-32, ura4-D18*	This study
YLJ228	*h-, NAT-nse3-R254E, mat1_m-cyhS, smt0, rpl42::cyhR (sP56Q), ade6-M210, leu1-32, ura4-D18*	This study
YLJ252	*h-, Δsgf11::G418, nse3-R254E::Lox, ura4-D18, ade6-704?, ade6-M216?, leu1-32*	This study
YLJ255	*h-, Δgcn5::G418, nse3-R254E::Lox, ura4-D18, ade6-704?, ade6-M216?, leu1-32*	This study
YLJ262	*h-, Δsgf29::G418, nse3-R254E::Lox, ura4-D18, ade6-704?, ade6-M210?, leu1-32*	This study
YLJ265	*h+, Δada2::G418, nse3-R254E::Lox, ura4-D18, ade6-704?, ade6-M216?, leu1-32*	This study
YLJ268	*h-, Δubp8::G418, nse3-R254E::Lox, ura4-D18, ade6-704?, ade6-M216?, leu1-32*	This study
YLJ272	*h-, Δngg1::G418, nse3-R254E::Lox, ura4-D18, ade6-704?, ade6-M216?, leu1-32*	This study
YLJ290	*h+, Δsgf73::G418, nse3-R254E::Lox, ura4-D18, ade6-704?, ade6-M216?, leu1-32*	This study
YLJ375	*h-, Δgcn5::G418, nse4-FLAG-His::KanR, ura4-D18, ade6-M216?, leu1-32*	This study
YLJ386	*h-, Δgcn5::G418, ura4-D18, ade6-704?, ade6-M216?, leu1-32*	This study
YLJ388	*h?, Δgcn5::G418, smc6-x, ura4-D18, ade6-704?, ade6-M216?, leu1-32*	This study
YLJ391	*h?, Δgcn5::G418, smc6-74, ura4-D18, ade6-704?, ade6-M216?, leu1-32*	This study
YLJ426	*h+, Δubp8::G418, nse4-FLAG-His::KanR, ade6-704?, ade6-M216?, leu1-32, ura4-D18?*	This study
YLJ459	*h-, Δtra1::G418, nse3-R254E::Lox, ura4-D18, ade6-704?, ade6-M216?, leu1-32*	This study
YLJ462	*h?, nse2-SA::URA4, Δgcn5::G418, ura4-D18, ade6-704?, ade6-M216?, leu1-32*	This study
YLJ496	*h-, Δada2::G418, nse4-FLAG-His::KanR, ade6-704?, ade6-M216?, leu1-32, ura4-D18*	This study
YLJ507	*h?, nse4-FLAG-His:KanR, gcn5-Myc13::natMX6, ura-D18, ade6-M216?, leu1-32?*	This study
YLJ510	*h?, nse4-FLAG-His:KanR, gcn5-E191Q-Myc13::natMX6, ura-D18, ade6-M216?, leu1-32?*	This study
YPK150	*h?, nse4:GFP::HphMx4, Δgcn5::G418, ade6-216?, ura4-D18?, leu1-32?*	This study

YPK165	*h?, nse4:GFP::HphMx4, Δbrc1::G418, leu1-32?, ura4-D18?*	This study

Table S6 - qPCR primers

vht1	I:4959888-4960015	prBS103	fw	5′ GAGGAGACAAAGTTTTCATCCATTTGGG 3′
		prBS104	rev	5′ GGGAGTCGTTGCTATTGCCC 3′
fip1	I:4233546-4233678	prBS105	fw	5′ CGACGTTCCTTGTACCACATGAGG 3′
		prBS106	rev	5′ GGTGGTTACATGATCTTCAATGCCA 3′
rps502	I:3496612-3496743	prBS113	fw	5′ GGAAAGCAGCCTCACGAGC 3′
		prBS114	rev	5′ GTCCTCGTGAAGACTCCACTCG 3′
ser3	III:470819-470947	prBS109	fw	5′ CGATAAAAAGCTCGTTAACTTGGCG 3′
		prBS110	rev	5′ GGTGCTGTTAACTTCCCTGAGG 3′
tef3	III:1688122-1688266	prBS111	fw	5′ GAGTGCTCTTTCCTCGTCGG 3′
		prBS112	rev	5′ CTCAGCACGGTCAACTTCAGC 3′
slx9	I:499186-499310	prBS129	fw	5′ CGGTAAATTGATACGCCC 3′
		prBS130	rev	5′ GACATCCCGACAATACATC 3′
